# Exercise Communication for Breast Cancer Survivors: A Systematic Scoping Review

**DOI:** 10.1001/jamanetworkopen.2025.8862

**Published:** 2025-05-16

**Authors:** Oliver W. A. Wilson, Kaitlyn M. Wojcik, Jinani Jayasekera, Laura Q. Rogers, Wendy Demark-Wahnefried, David Farrell, Gisela Butera, Charles E. Matthews, Richard L. Street

**Affiliations:** 1 Division of Intramural Research at the National Institute on Minority Health and Health Disparities, National Institutes of Health, Bethesda, Maryland; 2 Division of Preventive Medicine, Department of Medicine at University of Alabama at Birmingham; 3 Department of Nutrition Sciences at University of Alabama at Birmingham; 4 People Designs, Durham, North Carolina; 5 Office of Research Services, National Institutes of Health Library, Bethesda, Maryland; 6 Metabolic Epidemiology Branch at the National Cancer Institute, National Institutes of Health, Bethesda, Maryland; 7 Department of Communication and Journalism at Texas A & M University, College Station

## Abstract

**Importance:**

Exercise may offer various clinical benefits to breast cancer survivors. However, exercise participation among survivors is low. Discussions about exercise can increase participation, but details regarding implementation of exercise communication for breast cancer survivors are unclear.

**Objective:**

To examine the development and implementation of explicitly reported exercise communication for breast cancer survivors.

**Evidence Review:**

The Preferred Reporting Items for Systematic reviews and Meta-Analyses extension for Scoping Reviews was used. Six databases (PubMed/MEDLINE, Cochrane Library, Embase, Web of Science, Communication and Mass Media Complete, and PsycINFO) were searched for articles describing exercise communication for female breast cancer survivors in any geographical location or setting published up to April 2024. Information about study and participant characteristics, exercise communication, and intervention components and effects were extracted.

**Findings:**

Thirty-nine studies were included. Information consistent with exercise guidelines was communicated in 32 studies for aerobic exercise (82.1%) and 7 studies for muscle-strengthening exercise (17.9%). More studies communicated information about exercise benefits (20 [51.3%]) compared with exercise safety (3 [7.7%]). Studies included breast cancer survivors who were predominantly White (median [range], 85.5% [47.4%-98.7%]). Most included breast cancer survivors were educated (median percentage, 50.7% were college graduates) and had completed primary treatment (eg, median percentage, 66.7% had completed chemotherapy). Seventeen individualized (ie, tailored) information based on demographic or clinical characteristics (43.6%), and 5 considered contextual factors, such as the environment (12.8%). Most interventions reported an increase in exercise (19 of 21 [90.4%]). Heterogeneity in intervention design (eg, dietary advice or exercise trackers in addition to exercise information) and evaluation (eg, intervention length and outcomes) prevented quantitative synthesis of intervention effects.

**Conclusions and Relevance:**

In this systematic scoping review of exercise communication for breast cancer survivors, interventions including exercise communication appeared to show a favorable effect. However, data were limited about the development and implementation of these interventions among underrepresented breast cancer survivors. Interest in research involving exercise communication for breast cancer survivors in clinical settings remains strong.

## Key Points

**Question:**

What is the content, format, delivery, and impact of exercise-related information being communicated to breast cancer survivors?

**Findings:**

In this systematic scoping review of 39 studies, 32 studies communicated information consistent with aerobic exercise guidelines and 20 studies communicated about the benefits of exercise; however, only 7 studies communicated information about muscle-strengthening exercise and 3 communicated information about exercise safety. Exercise communication was associated with favorable outcomes, including increased exercise participation and quality of life among breast cancer survivors.

**Meaning:**

These findings suggest that exercise communication interventions may increase exercise participation and quality of life, however, a lack of individualization and omitting information on muscle-strengthening exercise, gradual progression, and safety may be limiting the outcomes of messages.

## Teaser

This systematic scoping review examines the development and implementation of explicitly reported exercise communication for breast cancer survivors.

## Introduction

There are approximately 4 million breast cancer survivors in the US,^1^ and less than half meet exercise recommendations (eg, 37.7% engage in aerobic exercise 150 minutes or more per week, and 17.6% engage in muscle-strengthening exercise 2 or more days per week).^2^ This could be due to several factors, such as cardiotoxicity from treatment,^3^ low social support, environmental factors (eg, access to recreational amenities), a lack of knowledge,^4,5^ and changing exercise preferences.^6^ As a result, many may be forgoing benefits associated with exercise, such as reducing the risk of fatigue,^7,8^ improving psychological well-being,^8^ and reducing the risk of recurrence^9,10,11^ and mortality.^11^ Discussions about exercise between health care clinicians and patients can achieve meaningful increases in exercise.^12,13^ Exercise discussions are considered an essential part of breast cancer survivorship care plans^14,15,16^ and are now necessary for national accreditation as a breast center in the US.^15^ Patient-clinician communication can also help address disparities.^17^ Yet, only 54.4% of US breast cancer survivors report having discussed exercise with a health care clinician^18^ due to, in part, a lack of knowledge, time, and self-efficacy.^19,20^

The Physical Activity Messaging Framework and Physical Activity Messaging Checklist (PAMC) were recently developed to create and evaluate communication of information regarding physical activity.^21^ The PAMC identifies content, format, and delivery as important components of exercise communication.^21^ Content encompasses characteristics, such as what to do (ie, recommendations on exercise quantity and type), why you should do it (ie, information about the benefits/risks of exercise), and the tailoring (ie, individualization) of communication for individual needs, values, and preferences. Format and delivery concern how information is conveyed (eg, written vs verbal) and by whom (ie, oncologists vs exercise specialists).^21^

Current cancer exercise guidelines recommend that cancer survivors receive individualized exercise prescriptions that gradually progress toward meeting national recommendations.^16,22,23^ However, they focus primarily on reviewing evidence for the efficacy of exercise for cancer survivors (predominantly breast cancer survivor research) and do not provide recommendations related to exercise message content (ie, what exercise to do and why), or how to individualize messages based on cancer survivors’ preferences and demographic, clinical, or treatment characteristics.^16,22,23^ They also do not outline how information should be delivered or by whom. Thus, at present, evidence about the implementation of exercise communication (ie, what exercise information is being communicated, how such information is being communicated, and by whom) for breast cancer survivors is limited. There is also limited information on the intervention effects on outcomes, such as psychological antecedents to exercise (eg, self-efficacy, intentions), exercise participation, and/or health outcomes (eg, quality of life,) attributable to exercise communication. Finally, among the few studies to have examined the exercise communication preferences of breast cancer survivors, each have reported that receiving exercise face-to-face (47.0% to 73.2%) and from exercise specialists (40.0% to 81.0%) are preferred over other formats and delivery methods.^6,24,25,26^

Cancer exercise guidelines offer limited advice about the content, format, and delivery of exercise information for cancer survivors.^16^ To our knowledge, there are no prior reviews that have synthesized the existing literature about exercise communication for cancer survivors. Because breast cancer is the most common cancer among women,^27^ this review focused on breast cancer survivors. An evaluation of existing evidence on the implementation of exercise programming for breast cancer survivors may help inform the development of novel strategies to communicate exercise information to breast cancer survivors in clinical settings,^28,29,30^ especially since most cancer survivors (82.2%) prefer health care clinicians to initiate exercise discussions.^31^ Therefore, the aims of this scoping review were to examine, map, and critically evaluate content, format, delivery, and outcomes of exercise communication for breast cancer survivors. For the purposes of this review, exercise communication was defined as reported communication on the amount, benefits, and/or risks of exercise.

## Methods

### Protocol and Registration

This review was conducted in accordance with the Preferred Reporting Items for Systematic Reviews and Meta-analyses for Scoping Reviews (PRISMA-ScR) reporting guideline^32^ and established methodological frameworks.^33,34^ It was registered in Open Science Framework.^35^

### Eligibility Criteria

The focus of this review was articles describing the development and/or delivery of exercise communication for female breast cancer survivors (ie, women diagnosed with breast cancer regardless of treatment status) in any geographical location or setting. Inclusion criteria included studies that included female breast cancer survivors, reported details about the development and/or delivery of exercise communication, were primary empirical research studies, and written in English. Exclusion criteria included studies that were not empirical research studies, not written in English, did not report details about the development and/or delivery of exercise communication, and did not include female breast cancer survivors. Inclusion and exclusion criteria are detailed further in the eMethods and eTable 1 in Supplement 1.

### Information Sources and Search Strategy

The comprehensive search strategy of 6 databases (ie, PubMed/MEDLINE, Cochrane Library, Embase, Web of Science, Communication and Mass Media Complete, and PsycINFO) included keywords related to physical activity, cancer survivorship, and counseling, messaging, or communication (eAppendix 1 in Supplement 1). We conducted a comprehensive search of the literature from the inception of each database until April 2024.

### Selection of Sources of Evidence

Covidence (Veritas Health Innovation, Melbourne, Australia) was used to complete the data screening process.^36^ Titles and abstracts, followed by full texts, were screened for eligibility. Disagreements were resolved via discussion.

### Data Charting Process and Items

Details about extracted information are reported in eTable 2 in Supplement 1. Study authors were contacted via email for additional information and clarification if information was missing or unclear. Findings are reported at the study level.^37^Data were analyzed using Excel version 16.95 (Microsoft).

### Synthesis of Results

Extracted information was summarized using a narrative approach, as outlined by Cochrane.^38^ Heterogeneity in intervention design (eg, dietary advice or fitness trackers in addition to exercise information) and evaluation (eg, intervention length and outcomes) limited our ability to conduct a quantitative synthesis of intervention effects across all studies. Therefore, a narrative approach was used to summarize information. Results were stratified into studies conducted within and outside of the US.

## Results

### Selection of Sources of Evidence

Initial searches retrieved 3193 sources after duplicate removal. Reports were screened at the title and abstract levels, followed by a full-text review of 552 remaining sources. Three additional articles were identified by references list searches. Thirty-nine studies comprising 70 articles were identified and proceeded to extraction (eFigure 1 in Supplement 1).^39,40,41,42,43,44,45,46,47,48,49,50,51,52,53,54,55,56,57,58,59,60,61,62,63,64,65,66,67,68,69,70,71,72,73,74,75,76,77,78,79,80,81,82,83,84,85,86,87,88,89,90,91,92,93,94,95,96,97,98,99,100,101,102,103,104,105,106,107,108^

### Source Characteristics

Source characteristics are summarized in eAppendix 2 in Supplement 1, along with survivor characteristics (demographic, clinical, and contextual), which are reported in detail in eTables 3 to 6 in Supplement 1. Most of the 39 studies were conducted in the US (21 studies [53.8%]), and 30 studies reported the results of a randomized clinical trial (RCT) or intervention.^40,42,43,46,47,48,49,50,52,58,59,60,61,62,63,65,66,67,68,69,70,71,72,73,74,75,76,77,78,80,81,82,83,84,85,91,98,99,106,107,108^ Twenty-five of the 70 articles were published between 2020 and 2024 (35.7%).^39,40,43,45,49,51,53,59,62,66,68,70,71,72,73,75,79,84,86,87,88,90,99,103,105^ Most of the 39 studies were conducted in the US (21 [53.8%]), 25 studies were published from 2020 to 2024 (64.1%), and 30 studies reported the results of a randomized clinical trial (RCT) or intervention (76.9%).^42,43,44,46,47,48,50,52,53,54,55,56,57,58,59,63,64,65,66,67,68,69,70,71,72,73,74,75,76,77,78,79,80,81,82,83,85,86,87,89,90,91,92,93,94,95,96,97,98,99,100,101,102,103,104,106,107,108^ Survivors had a median (range) age of 55.5 (41.0-67.0) years and were predominantly White females (median [range], 85.5% [47.4%-98.7%]). The survivors were predominantly college educated (median percentage, 50.7%), resided in urban areas,^69,70,71,72,73,74,75,76,77,97,100,101,102,105,108^ and had completed primary treatment (eg, median percentage, 66.7% had completed chemotherapy). Table 1 summarizes intervention design characteristics. Full details on intervention duration, follow-up period, theoretical framework, and nonexercise communication intervention components are reported in eTable 7 in Supplement 1.

**Table 1.** Summary of Intervention Design Characteristics

Intervention component	Studies, No. (%)		
	US	Non-US	Total
Theoretical framework			
Social cognitive theory	13 (61.9)	4 (22.2)	17 (43.6)
Transtheoretical model	3 (14.3)	3 (16.7)	6 (15.4)
Theory of planned behavior	1 (4.8)	4 (22.2)	5 (12.8)
Other	2 (9.5)	1 (5.6)	3 (7.7)
No theory reported	6 (28.6)	7 (38.9)	13 (33.3)
Information communicated			
Exercise only	7 (33.3)	10 (55.6)	17 (43.6)
Exercise + diet	11 (52.4)	6 (33.3)	18 (46.2)
Exercise + weight management	4 (19.0)	1 (5.6)	5 (12.8)
Exercise + treatment and related adverse effects	1 (4.8)	3 (16.7)	4 (10.3)
Exercise + health behaviors	1 (4.8)	2 (11.1)	3 (7.7)
Exercise + well-being	1 (4.8)	1 (5.6)	2 (5.1)
Exercise + general breast cancer information	0	2 (11.1)	2 (5.1)
Other, noninformation communication, intervention component(s)			
Exercise tracker (ie, pedometer or exercise watch)	11 (52.4)	8 (44.4)	19 (50.0)
Exercise log	4 (19.0)	2 (11.1)	6 (15.4)
Mobile application	1 (4.8)	2 (11.1)	3 (7.7)
Scale to measure weight	2 (9.5)	1 (5.6)	3 (7.7)
Other	1 (4.8)	4 (22.2)	5 (12.8)
No nonexercise information communication component	8 (38.1)	8 (44.4)	16 (41.0)

#### Intervention Duration and Follow-Up Period

The median (IQR) intervention duration was 14 (12-26) weeks (34 studies [87.2%]),^40,42,43,44,46,47,48,49,50,51,52,53,54,55,56,57,58,59,63,64,65,66,67,68,69,70,71,72,73,74,75,76,77,78,79,80,81,82,83,84,85,86,87,88,89,90,91,92,93,94,95,96,97,98,99,100,101,102,103,104,106,107,108^ with the duration ranging from 0 days (a 1-off intervention) to 4 years. The median (IQR) follow-up period was also 12 (12-26) weeks (11 studies [28.2%]), with the period ranging from 12 weeks to 1 year.

#### Theoretical Framework

Social cognitive theory was the most commonly used theory (17 studies [43.6%]).^40,42,43,44,45,46,47,48,49,50,52,59,60,61,65,66,67,68,69,70,71,72,73,74,75,76,77,79,80,81,82,83,84,86,87,89,90,91,99,100,101,102,107^ The transtheoretical model (6 studies [15.4%])^41,47,50,53,57,65,69,70,71,72,73,74,75,76,77^ or theory of planned behavior (5 studies [12.8%])^41,85,94,95,96,97,104^ were also mentioned in multiple studies. Thirteen studies did not report a theoretical framework (33.3%).^39,51,54,55,56,58,62,63,64,78,88,92,93,105,106,108^ Social cognitive theory was used by 13 of 21 US-based studies (61.9%)^40,42,43,46,47,48,49,50,52,59,60,61,65,66,67,68,69,70,71,72,73,74,75,76,77,80,81,82,83,84,91,99^ compared with 4 of 18 non-US based studies (22.2%)^44,45,79,86,87,89,90,100,101,102^ (Table 1).

#### Intervention Components

Seventeen of 30 studies reported only communicating about exercise.^41,46,47,48,49,50,51,53,56,58,59,64,65,66,67,68,69,70,71,72,73,74,75,76,77,88,94,95,96,97,99,100,101,102,104,105^ An exclusive focus on exercise was more common among non-US studies compared with US studies. Among studies that communicated information on another topic, diet was the most common, followed by weight management and cancer treatment and related adverse effects. Compared with non-US studies, more US studies communicated information regarding diet and weight management. Twenty-four studies reported having an intervention component beyond communication designed to affect exercise or related outcomes (61.5%).^41,43,44,45,46,47,48,49,50,51,52,53,56,58,59,64,66,67,68,78,79,80,81,85,88,89,90,92,93,94,95,96,97,98,99,105,106,107,108^ Providing survivors with an exercise tracker (eg, pedometer) was the most common. Other components reported by multiple studies included an application accompanying the exercise tracker, a scale to measure weight, and providing exercise equipment.

### Content of Exercise Information Communicated to Breast Cancer Survivors

Details regarding what exercise to do (ie, recommendations on quantity and type of exercise), why to do exercise (ie, information about the benefits or risks of exercise), and individualization are summarized in Table 2. Full details on the content of exercise information communicated to survivors are reported in eTables 8 and 9 in Supplement 1.

**Table 2.** Summary of Exercise Information Content Communicated to Breast Cancer Survivors^a^

Exercise communication characteristic	Studies, No. (%)		
	US	Non-US	Total
What exercise to do			
Aerobic exercise			
≥150 min per wk of moderate-intensity exercise	15 (71.4)	10 (55.5)	25 (64.1)
Approximately 10 000 steps per wk	5 (23.8)	6 (33.3)	11 (28.2)
Other	4 (19.0)	5 (27.8)	9 (23.1)
Muscle-strengthening exercise			
Approximately 2 sessions per wk	3 (14.3)	4 (22.2)	7 (17.9)
Mentioned, not specified	0	2 (11.1)	2 (5.1)
Not reported	18 (85.7)	12 (66.7)	30 (76.9)
Gradual progression			
Mentioned for aerobic exercise	5 (23.8)	1 (5.6)	6 (15.4)
Mentioned for muscle-strengthening exercise	0	1 (5.6)	1 (2.6)
Why to do exercise			
Exercise benefits communicated to survivors			
Mental well-being, fun, and happiness	3 (14.3)	6 (33.3)	9 (23.1)
Fitness or reduced fatigue or tiredness	1 (4.8)	5 (27.8)	6 (15.4)
Decreased risk of recurrence	1 (4.8)	4 (22.2)	5 (12.8)
Decreased risk of mortality	0	3 (16.7)	3 (7.7)
Decreased pain	1 (4.8)	1 (5.6)	2 (5.1)
Weight management	2 (9.5)	0	2 (5.1)
Physical functioning or well-being	1 (4.8)	1 (5.6)	2 (5.1)
Self-esteem or image	1 (4.8)	1 (5.6)	2 (5.1)
Other benefits	2 (9.5)	1 (5.6)	3 (7.7)
Benefits mentioned, but not specified	2 (9.5)	3 (16.7)	5 (12.8)
No report of communicating benefits	12 (57.6)	9 (50.0)	21 (53.8)
Why exercise was communicated to survivors			
Weight management	9 (42.9)	3 (16.7)	12 (30.8)
Increasing exercise levels	2 (9.5)	5 (27.8)	7 (17.9)
Improving quality of life	1 (4.8)	3 (16.7)	4 (10.3)
Improving cognitive function	2 (9.5)	0	2 (5.1)
Other reasons	2 (9.5)	1 (5.6)	3 (7.7)
No report of why exercise was communicated	5 (23.8)	7 (38.9)	12 (30.8)
Exercise safety information communicated to survivors			
Yes	1 (4.8)	1 (5.6)	2 (5.1)
No	20 (95.2)	17 (94.4)	37 (94.9)
Individualization and considerations			
Individualization mentioned	10 (47.6)	7 (38.9)	17 (43.6)
Considerations mentioned	3 (14.3)	2 (11.1)	5 (12.8)
No individualization or considerations mentioned	10 (47.6)	10 (55.6)	20 (51.3)
Source			
American College of Sports Medicine	3 (14.3)	3 (16.7)	6 (15.4)
American Cancer Society	5 (23.8)	1 (5.6)	6 (15.4)
National or federal guidelines	2 (9.5)	2 (11.1)	4 (10.3)
Multidisciplinary Roundtable^b^	0	3 (16.7)	3 (7.7)
World Health Organization	0	2 (11.1)	2 (5.1)
World Cancer Research Fund or American Institute for Cancer Research	1 (4.8)	1 (5.6)	2 (5.1)
Other	4 (19.0)	1 (5.6)	5 (12.8)
No source	7 (33.3)	8 (44.4)	15 (38.5)

^a^ Numbers within the table sections may sum to more than 38 because some studies reported multiple benefits, reasons, and sources.

^b^ Endorsed by organizations, such as American Cancer Society, American College of Sports Medicine, and the US Centers for Disease Control and Prevention.^16^

#### What Exercise to Do

Every study communicated information about aerobic exercise (39 [100.0%]),^39,40,41,42,43,44,45,46,47,48,49,50,51,52,53,54,55,56,57,58,59,60,61,62,63,64,65,66,67,68,69,70,71,72,73,74,75,76,77,78,79,80,81,82,83,84,85,86,87,88,89,90,91,92,93,94,95,96,97,98,99,100,101,102,103,104,105,106,107,108^ compared with 9 that also communicated information on muscle-strengthening exercise (23.1%). Thirty-two studies (82.1%) communicated guideline-recommended aerobic exercise information (ie, the equivalent of approximately 150 or more minutes per week of moderate-intensity exercise and/or 10 000 or more steps per week^109^),^39,40,41,42,43,44,45,46,47,48,49,50,51,52,53,54,55,56,57,58,59,60,61,62,63,64,65,66,67,68,69,70,71,72,73,74,75,76,77,78,79,80,81,82,83,84,85,86,87,88,89,90,91,92,93,94,95,96,97,98,99,107^ compared with 7 studies (17.9%) that communicated guideline-recommended muscle-strengthening exercise information (ie, approximately 2 days/week^109^).^42,44,45,52,56,64,79,80,81,89,90,91,100,101,102,103,104^ The publication of studies communicating muscle-strengthening exercise information were distributed relatively evenly between 2007 and 2024. Gradual progression was mentioned in 6 studies for aerobic exercise (15.4%),^52,62,66,67,68,80,81,85,98,99,103^ and 1 for muscle-strengthening (2.6%).^103^ A greater proportion of US studies communicated information aligning with aerobic exercise guidelines and mentioned gradual progression in relation to aerobic exercise. By contrast, more non-US studies communicated information approximating muscle-strengthening exercise guidelines.

#### Why to Do Exercise

Eighteen studies communicated exercise benefits to survivors (46.2%).^41,46,51,53,56,58,59,60,61,62,63,64,65,66,67,68,69,70,71,72,73,74,75,76,77,88,94,95,96,97,99,100,101,102,104,105^ Benefits centered around mental well-being, fun, and happiness as the most common, followed by improved fitness or reduced fatigue or tiredness, and risk of recurrence. Twenty-seven studies reported why exercise information was communicated to survivors (69.2%).^39,40,42,43,44,45,46,48,49,50,52,54,55,57,58,59,60,61,62,66,67,68,69,70,71,72,73,74,75,76,77,79,80,81,84,85,86,87,88,89,90,91,94,95,96,97,98,99,100,101,102,103,106,107^ The most common reason was weight management, followed by increasing exercise levels, quality of life, and cognitive function. Non-US studies mentioned benefits more frequently compared with US studies, with the exception of weight management. In contrast to exercise benefits, 3 studies reported communicating information on exercise safety (7.7%).^98,103,104^ One study informed survivors that there was no risk of lymphedema if survivors were supervised when exercising,^104^ and the other study provided information on exercising safely unsupervised.^98^

#### Individualization and Considerations

Seventeen studies (43.6%)^41,42,44,45,47,48,49,50,51,52,53,56,62,64,66,67,68,69,70,71,72,73,74,75,76,77,78,80,81,85,91,98,99,100,101,102,103^ mentioned individualizing exercise communication (eg, tailoring communication based on demographic or clinical characteristics), though how communication was individualized was often not reported in much detail. Five other studies reported making considerations when communicating exercise to breast cancer survivors based on factors, such as a survivor’s values, enjoyment, social support, access to resources, and environment (12.8%).^52,70,80,81,85,103^ Details on considerations made by these studies are reported in eAppendix 3 in Supplement 1.

#### Sources Cited

Most studies offered a citation of the source underpinning the exercise information communicated (Table 2 and eTable 8 in Supplement 1). The most common guidelines cited were the American College of Sports Medicine (ACSM)^110,111,112^ (7 studies[17.9%])^47,48,49,50,53,79,88,89,90,103^ and American Cancer Society (ACS)^113,114^ (6 studies [15.4%]),^40,42,46,57,59,82,83,84,85,91^ followed by national or federal guidelines^115,116^ (3 studies [7.7%])^54,55,69,70,71,72,73,74,75,76,77,100,101,102^ and the 2019 International Multidisciplinary Roundtable^16^ (3 studies [7.7%]),^51,88,103^ which were endorsed by organizations, such as ACS, ACSM, and the US Centers for Disease Control and Prevention (CDC). World Health Organization guidelines^117^ (2 studies [5.1%])^39,86,87^ and World Cancer Research Fund/American Institute for Cancer Research guidelines^118^ were also cited (2 studies [5.1%]).^40,79,84,89,90^

### Format and Delivery of Exercise Information Communicated to Breast Cancer Survivors

The format of information delivery and by whom are summarized in Table 3 (eTable 10 in Supplement 1 for full details on format and delivery). Sixteen studies used both written and verbal communication (41.0%),^40,44,45,46,52,53,54,55,59,62,66,67,68,69,70,71,72,73,74,75,76,77,79,80,81,82,83,84,88,89,90,98,99,103,104,107^ 11 used written alone (28.2%),^39,41,42,43,60,61,63,86,87,91,94,95,96,97,100,101,102,106^ 5 used verbal alone (12.8%),^47,48,49,50,58,85,108^ and 7 did not report the methods (18.9%).^51,56,57,64,65,78,92,93,105^

**Table 3.** Summary of Format and Delivery of Exercise Information Communicated to Breast Cancer Survivors^a^

Exercise communication characteristic	Studies, No. (%)		
	US	Non-US	Total
Written communication			
Format			
Tangible printed materials (eg, handout)	11 (52.4)	7 (38.9)	18 (46.2)
Text or email	4 (19.0)	3 (16.7)	7 (17.9)
App or website	2 (9.5)	3 (16.7)	5 (12.8)
No written communication reported	7 (33.3)	5 (27.8)	12 (30.8)
Delivery			
Researchers	2 (14.3)	1 (7.7)	3 (11.1)
Medical staff	0	1 (7.7)	1 (3.7)
No details on written delivery reported	12 (85.7)	11 (84.6)	23 (85.2)
Verbal communication			
Format			
Telephone	10 (47.6)	3 (16.7)	13 (33.3)
Face-to-face (ie, in person)^b^	7 (33.3)	4 (22.2)	11 (28.2)
Video conference	0	1 (5.6)	1 (2.6)
Videotape	1 (4.8)	0	1 (2.6)
No verbal communication reported	7 (33.3)	11 (61.1)	18 (46.2)
Delivery			
Researcher	3 (21.4)	3 (42.9)	6 (28.6)
Coach	3 (21.4)	1 (14.3)	4 (19.0)
Qualified exercise professional	1 (7.1)	2 (28.6)	3 (14.3)
Dietitian	1 (7.1)	1 (14.3)	2 (9.5)
Peer partner	0	1 (14.3)	1 (4.8)
Clinical psychologist	1 (7.1)	0	1 (4.8)
Physician, nurse practitioner, or physician assistant	1 (7.1)	0	1 (4.8)
No details on verbal delivery reported	4 (28.6)	0	4 (19.0)

^a^ Percentages for delivery were calculated using the number of studies to report using the format as the denominator.

^b^ All face-to-face communication took place in clinical settings.

#### Written Communication

Among the 27 studies that reported communicating written information (69.2%),^39,40,41,42,43,44,45,46,52,53,54,55,59,60,61,62,63,66,67,68,69,70,71,72,73,74,75,76,77,79,80,81,82,83,84,86,87,88,89,90,91,94,95,96,97,98,99,100,101,102,103,104,106,107^ the most common method was tangible printed materials (eg, handout, guidebook), followed by text or email and an app or website (eTable 10 in Supplement 1). Tangible print materials were more commonly used in US studies compared with non-US studies. Four studies reported who communicated written information (10.3%),^41,60,61,86,87,98^ with researchers being the most common.

#### Verbal Communication

Among the 21 studies that reported communicating information verbally (53.8%),^40,44,45,46,47,48,49,50,52,53,54,55,58,59,62,66,67,68,69,70,71,72,73,74,75,76,77,79,80,81,82,83,84,85,88,89,90,98,99,103,104,107,108^ the telephone was the most common method, followed by face-to-face (ie, in person; all in clinical settings), and videoconference (eTable 10 in Supplement 1). Verbal communication was more common in US studies than non-US studies. Researchers were the most common reported communicators, followed by coaches, and qualified exercise professionals.

#### Language and Other Information

Two studies reported exercise communication in a language alongside English—1 Canadian that included French (5.1%)^44,45^ and 1 in the US that included Spanish (2.6%).^40,84^ One study that did not offer information on the communication method reported that physical therapists communicated information (2.6%).^56,64^

### Intervention Outcomes

Ten studies did not evaluate effects of exercise communication on psychological antecedents to exercise, exercise participation, and/or outcomes associated with exercise because they were either cross-sectional, reported methods of a forthcoming intervention, or focused on evaluating website/application usability (25.6%).^39,40,41,49,51,60,61,62,84,88,105,106^ Intervention outcomes and effects are summarized in Table 4 and further details are reported in eTable 11 in Supplement 1.

**Table 4.** Summary of Intervention Outcomes and Effects^a^

Outcomes	Effects, No. of studies		
	Favorable	None	Unfavorable
Psychological antecedents of exercise			
Exercise self-efficacy	2	NR	NR
Intention to exercise	1	NR	NR
Motivational readiness to exercise	1	NR	NR
Attitudes toward exercise	1	NR	NR
Exercise planning	1	NR	NR
Social support for exercise	NR	1	NR
Exercise participation	19	1	NR
Quality of life	8	NR	NR
Physical quality of life	2	NR	NR
Mental quality of life	1	1	NR
Physical health			
Anthropometric characteristics (eg, BMI)	10	2	NR
Physical functioning	6	NR	NR
Fatigue	5	NR	NR
Fitness	4		
Metabolic risk factors (eg, blood pressure)	4	NR	NR
Pain	1	NR	NR
Grip strength	1	NR	NR
Disease-free survival	1	NR	NR
Serologic indices	NR	1	NR
Mental health	1	1	NR
Anxiety, depression, and mood	3	1	NR
Body image	1	NR	NR
Psychological menopause symptoms (eg, nervousness)	1	NR	NR
Sleep-related impairment	1	NR	NR

Abbreviations: BMI, body mass index; NR, not reported.

^a^ Ten studies did not evaluate the effects of interventions; some studies report effects pertaining to multiple outcomes.

#### Psychological Antecedents to Exercise

Two studies reported improved exercise-related efficacy^57,86,87^ (Table 4 and eTable 11 in Supplement 1). Improvements in motivational exercise intentions, motivational readiness, attitudes towards exercise, and exercise planning were also reported. One reported no change in social support for exercise.^43^ Intention and planning were found to partially mediate the effects of the intervention on exercise.^94,95,96^ Another found that beliefs about benefits and risks of exercise were more favorable among those who received expert exercise advice rather than via a breast cancer survivor testimonial.^104^

#### Exercise Participation

Nineteen studies reported an increase in aerobic exercise.^42,52,53,54,55,56,57,58,63,64,65,66,67,68,78,80,81,82,83,85,86,87,91,94,95,96,97,98,99,100,101,102,103^ However, 1 that communicated information at a single time point and followed up 4 weeks and 12 weeks later reported change.^94,95,96^ Four found that exercise communication plus an exercise tracker increased exercise more than information alone,^42,53,56,64,85,91^ whereas another found that adding a tracker made no difference.^97^ Only 1 examined the effects on muscle-strengthening exercise, reporting an increase.^100,101,102^

#### Quality of Life (QOL)

QOL improved in 8 studies^43,48,54,55,58,59,82,83,92,93^ that used the European Organization for Research and Treatment of Cancer (3 studies),^119,120^ Functional Assessment of Cancer Therapy- Breast (FACT-B) scale^121^ (3 studies), Adult QOL in Adult Cancer Survivors Scale (1 study),^122^ Patient-Reported Outcome Measurement Information System Global Health Scale (1 study),^123^ and Short Form Health Survey 36 (1 study).^124^ Seven of these studies targeted multiple factors associated with QOL by communicating information beyond just exercise.^43,48,49,54,55,58,82,83,92,93^

#### Physical Health

Anthropometric characteristics improved in 10 studies^43,46,52,54,55,59,69,70,71,72,73,74,75,76,77,78,79,80,81,86,87,89,90,92,93,103^ compared with 2 that reported no change.^58,86,87^ One study reported that extra counseling and tracking behavior did not increase weight loss.^46,59^ All of these studies communicated information relating to diet and/or weight management in addition to exercise information. Favorable intervention effects associated with physical functioning, fatigue, fitness, and metabolic risk factors (eg, blood pressure) were reported in multiple studies. Other favorable intervention effects reported by single studies pertained to pain, grip strength, and disease-free survival. Those who received interventions in addition to information had approximately 4% greater 5-year disease-free survival compared with those who received information only.^44,45^

#### Mental Health

Three studies reported improvements in anxiety, depression, and/or mood^47,48,50,66,67,68,69,70,71,72,73,74,75,76,77,99^ compared with 1 that reported no change.^107^ Other improvements reported included body image, psychological menopause symptoms, and sleep-related impairment.

### Key Findings and Knowledge Gaps

Key findings, along with gaps in knowledge relating to research and/or clinical practice, are summarized in Table 5. How exercise information is communicated to underrepresented breast cancer survivors is largely unknown, because participants were predominantly Non-Hispanic White, educated, resided in urban areas, and had completed primary treatments. Most studies were conducted in clinical settings and in North America, so it is also currently unclear whether the effects reported in the studies included in this review are the same among more diverse breast cancer survivors in different contexts. Despite more than a third of articles being published within the past 5 years, contemporary cancer exercise guidelines did not inform communication in most. Although the average intervention duration was approximately 3 months, a minority of studies included a follow-up period. This means there is a need for more longitudinal research evaluating the long-term and sustained effects of exercise. The majority of studies were underpinned by a theoretical framework. Yet, it is unknown how exercise communication influences the psychological constructs underpinning these frameworks and how these constructs are associated with exercise and related outcomes. Most interventions combine exercise communication with communication on other health behaviors (eg, diet) and/or other exercise promotion components (eg, fitness trackers). As such, it is unclear what effects are attributable specifically to exercise information communication. Most studies communicate aerobic exercise information consistent with recommendations, but a minority mention muscle-strengthening exercise. It is unclear how to individualize information based on demographic, clinical, and contextual characteristics. There were limited data to assess cultural relevance and safety of exercise messages. Exercise information is communicated both verbally and in writing using various methods and by various parties. However, who is best, willing, and able to communicate exercise information, and how to communicate such information has yet to be established.

**Table 5.** Key Findings and Related Gaps

Aspect	Key findings	Knowledge gaps
Target population	BCS were predominantly White, educated, resided in urban areas, and had completed primary treatment	How and what exercise information is communicated to underrepresented BCS is largely unknown
Research location or setting	Most studies are conducted in North America; most exercise communication is delivered in clinical settings	Implementation of exercise communication for BCS outside of North America, particularly in non-English speaking countries, and BCS outside of clinical settings (eg, in the community) is unclear
Year of publication	More than one-third (35.7%) of the articles included were published in the previous 5 years	Often the most recent cancer exercise guidelines were not drawn upon when communication exercise information
Study design	The median (IQR) intervention duration was 14 (12-26) wk; most studies did not include a follow-up period	Longitudinal evaluation of the long-term and sustained effects of exercise information communication is lacking
Theoretical framework	Most interventions were underpinned by a theoretical framework; the effect of interventions on psychological antecedents of exercise behavior were scarcely examined	How exercise communication influences the psychological constructs underpinning theoretical frameworks being used, and how these constructs (exercise antecedents) are associated with exercise participation and related outcomes is largely unknown
Intervention components	Most interventions communicate information beyond just exercise; most interventions include components beyond communication to promote exercise (eg, exercise trackers)	It remains unclear what effects are attributable to exercise information communication vs communication of information about other health behaviors (eg, diet) and/or other exercise promotion components (eg, fitness trackers)
Aerobic exercise	Most studies communicate aerobic exercise information consistent with recommendations	Gradual progression of aerobic exercise is not communicated consistently
Muscle-strengthening exercise	Few studies mention muscle-strengthening exercise	Muscle-strengthening exercise is not communicated consistently
Individualization	Less than half of studies individualize (ie, tailor) information	It is unclear how to individualize exercise communication based on demographic, clinical, and contextual characteristics; it is unclear how to ensure exercise communication is culturally safe and tailored
Format and delivery	Written communication is most common, but the author(s) of information is typically not reported; verbal communication is less common, but who communicates verbal information more commonly reported, and all face-to-face communication appears to occur in clinical settings	It has yet to be established: (1) who is best, willing, able to communicate exercise information; (2) how to communicate such information; (3) where survivors prefer to receive information
Effects and outcomes	Interventions including exercise communication appear to have a favorable effect on various outcomes	It is unclear whether the effects reported in the studies included in this review will be evident in more diverse BCS in different contexts

Abbreviation: BCS, breast cancer survivors.

## Discussion

This systematic scoping review examined the implementation and outcomes of exercise communication for breast cancer survivors. The trajectory of the number of articles published reporting exercise communication for breast cancer survivors showed that there is strong interest in research involving such communication. Information about the amount of aerobic exercise communicated to breast cancer survivors tended to be based upon current exercise guidelines.^16,22,23,109^ Similar to the wider body of exercise oncology literature,^125^ greater emphasis was given to aerobic exercise compared with muscle-strengthening exercise. Muscle-strengthening exercise was not a part of the initial cancer exercise guidelines published by the ACS in 2006,^113^ but was included in the 2008 US Department of Health and Human Services guidelines for adults,^126^ and the 2010 ACSM^111^ and 2012 ACS^114^guidelines for cancer exercise guidelines. However, incorporation of muscle-strengthening exercise into these guidelines did not appear to have resulted in a noticeable update of muscle-strengthening exercise into communication to breast cancer survivors.

The differences across studies conducted within and outside of the US highlight the need to consider the impact of system-level factors on exercise communication. The National Institute on Minority Health and Health Disparities research framework outlines how behavioral and health care system domains interact with individual, interpersonal, community, and societal factors to determine health outcomes.^127^ For instance, at an individual level, health literacy may be associated with exercise participation among breast cancer survivors.^128,129,130^ Interpersonal factors, such as patient-clinician relationships, may influence exercise self-efficacy.^60,131^ Physical and built environmental determinants could guide delivery of information on accessible places for survivors to safely engage in exercise within their communities.^132,133^ Finally, exercise referral and payment policies may also guide advice on where and how survivors could engage in exercise.^134^ However, a limited number of studies reported considering the influence of broader interpersonal, community, and societal factors on exercise communication. Similarly, few studies considered the framing, frequency, and tone of exercise messages.^21^

It is promising that many studies reported efforts to individualize communication, but exactly how communication was individualized remains largely unclear. Further research is needed to understand the priorities and concerns of breast cancer survivors to support individualized and culturally sensitive communication interventions.^135,136^ Although information concerning duration was consistently reported, the concept of gradual progression toward national exercise guidelines^109^ was reportedly communicated in only a small number of studies despite also being a part of cancer exercise guidelines published and/or endorsed by the ACS, ACSM, American Society of Clinical Oncology, and CDC.^16,22,23^ This apparent gap may be because progression is an essential element of any exercise prescription, and authors may have considered gradual progression as implicit. Gradual progression is important because most cancer survivors prefer to build up the amount of exercise they participate in and receive reassurance about exercise safety.^31^ This is, in part, because individuals with long-term conditions, such as breast cancer, tend to have high perceived concerns about the safety of exercise despite the actual risks being relatively low.^137^ However, similar to the lack of communication on gradual progression, information on exercise safety was scarcely reported to survivors, especially compared with the frequency of communicating benefits. Immediate short-term benefits (eg, mental well-being, reduced fatigue) were most commonly communicated, which is supported by current evidence.^138^ More distal long-term benefits, such as weight loss maintenance and mortality, were mentioned, too. Beyond the guidebook developed by Vallance et al,^139^ which was cited by several studies, no common information source of exercise benefits or risks emerged.

Regarding the format and delivery of communication, half of the studies used verbal communication and more than half used written communication. The authors of written communication and the setting in which it was delivered was largely unreported. By contrast, a variety of professions reportedly delivered verbal information, with allied health care clinicians communicating exercise information in only 2 studies. This is unsurprising because these discussions are not yet common practice in clinical settings,^18,140,141^ yet face-to-face communication was only reported to occur in clinical settings. Exercise promotion education among allied health care clinicians was relatively uncommon,^142,143^ which likely contributes to a lack of knowledge and self-efficacy to discuss exercise with survivors.^19^ Among breast oncologists, 1.2% considered their profession was most suited to promote exercise, with nurses (50.0%), physical therapists (33.3%), and other health professionals, such as exercise specialists, (11.2%) considered better suited.^141^ Further work is required to understand which professions within the health care system, and perhaps beyond, are both willing and able to take responsibility for different components of exercise promotion among cancer survivors. Although this may differ based on a variety of factors such as the clinic characteristics, available resources, and the formal or continuing education in exercise promotion that professionals have received.

The communication of information on other topics beyond exercise (eg, diet) and/or the use of other intervention components that may impact exercise (eg, exercise trackers) hampered the ability to draw concrete conclusions about the effects on exercise and related outcomes attributable to communicating exercise information. Findings suggest that communication of information about exercise may increase exercise and improve a range of outcomes including QOL, anthropometric variables, metabolic risk factors, fatigue, and mental health. Four studies suggested that pairing exercise information with an exercise tracker may increase exercise more than information alone.^42,53,56,64,85,91^ Future research is needed to establish what content, format, delivery, and framing are most effective (and for whom) and what other interventions components should be combined with the communication of exercise information to achieve the best outcomes. Most studies were underpinned by a theoretical framework, however, the effect of exercise communication on psychological antecedents of exercise was scarcely examined.

### Limitations

This review has limitations. First, reporting of exercise communication characteristics is a major limitation. As reported, information was missing about content, format, and delivery for many studies. Shortcomings in reporting also precluded the ability to extract information on other characteristics of communication content (eg, practical or supportive information on how to do exercise), framing (eg, gain vs loss), or tone (eg, formal, encouraging, or threatening), as well as cultural relevance and safety. Details about the volume or length (eg, words or duration), frequency, timing, and dosage of communication (ie, how often, at what time, and for how long) were reported infrequently.^21^ We excluded 51 articles because authors did not explicitly report whether information concerning the amount, benefits, and/or risks of exercise were communicated to survivors. Similarly, many studies were missing data on participant demographic, clinical, or contextual characteristics. For example, some studies did not report which racial and/or ethnic groups comprised the other category. In the future, there is a need for researchers to report on all components of the exercise messaging framework,^21^ as well as survivor characteristics. Our review is also limited by restricting it to articles with full text available in English, and thus evidence about exercise communication to breast cancer survivors in other languages may be missing. In addition, heterogeneity limited our ability to conduct a quantitative synthesis of intervention effects across all studies. Finally, these findings are largely based off articles comprised of survivors who are mostly aged 60 years or younger, non-Hispanic White, diagnosed with stage I or II cancer, college educated, postmenopause, who have completed treatment, and reside in urban areas. As a result, findings may not be generalizable to breast cancer survivors as a whole. Larger studies of survivors from different populations examining the outcomes of exercise communication are needed.

## Conclusions

To our knowledge, this is the first study to synthesize the literature concerning content, format, delivery, and outcomes of exercise communication for breast cancer survivors. Further formative research is needed to establish and coproduce the content, format, and delivery most likely to resonate with breast cancer survivors.^144^ Cancer exercise guidelines offer clear recommendations to individualize exercise prescriptions that gradually progress the amount of aerobic and muscle-strengthening exercise toward national recommendations. While aerobic exercise information was mostly consistent with national exercise guidelines, incorporating the concept of gradual progression and information on muscle-strengthening into messages that are individualized to individual needs has the potential to gain higher levels of receptivity. The format and delivery of exercise information varied greatly, despite breast cancer survivors appearing to prefer receiving information face-to-face and from exercise specialists.^6,24,25,26^ Clarity about who is best suited and able to deliver such information is needed to deliver consistent, and ultimately effective, information about exercise to breast cancer survivors. Future research should examine what content, format, delivery, and framing are most effective and how they can be individualized. Further research is needed to facilitate communication of individualized exercise recommendations to breast cancer survivors that: (1) encompasses gradual progression of both aerobic and muscle-strengthening exercise; (2) is individualized based on each survivor’s individual, clinical, and contextual characteristics; and (3) includes details on both the risks and benefits of exercise.

## Notes

Supplement 1. **eAppendix 1.** Search Strategies
eMethods.
**eTable 1. **Inclusion and Exclusion Criteria for the Scoping Review **eTable 2.** Information Extracted for This Scoping Review **eFigure 1.** Article Identification Process Using PRISMA Research Framework145 **eAppendix 3.** Study and Breast Cancer Survivor Characteristics **eTable 3.** Participant Characteristics **eTable 4.** Participant Demographic Characteristics **eTable 5.** Participant Clinical Characteristics **eTable 6.** Participant Contextual Characteristics **eTable 7.** Intervention Characteristics and Nonexercise Communication Components **eTable 8.** Content of Exercise Information Communicated to Breast Cancer Survivors **eTable 9.** Why Exercise Information Was Communicated **eAppendix 4.** Other Exercise Information Content **eTable 10.** Format and Delivery of Exercise Information Communicated to Breast Cancer Survivors **eTable 11.** Intervention Outcomes and Effects
eReferences.

Supplement 2.
Data Sharing Statement
