# Supplementary material for: Exercise Communication for Breast Cancer Survivors: A Systematic Scoping Review
Source: JAMA Netw Open. 2025 May 16;8(5):e258862. doi: 10.1001/jamanetworkopen.2025.8862 (PMC12084847; doi:10.1001/jamanetworkopen.2025.8862)
Supplement: Supplement 1. — eAppendix 1. Search Strategies eMethods. eTable 1. Inclusion and Exclusion Criteria for the Scoping Review eTable 2. Information Extracted for This Scoping Review eFigure 1. Article Identification Process Using PRISMA Research Framework145 eAppendix 3. Study and Breast Cancer Survivor Characteristics eTable 3. Participant Characteristics eTable 4. Participant Demographic Characteristics eTable 5. Participant Clinical Characteristics eTable 6. Participant Contextual Characteristics eTable 7. Intervention Characteristics and Nonexercise Communication Components eTable 8. Content of Exercise Information Communicated to Breast Cancer Survivors eTable 9. Why Exercise Information Was Communicated eAppendix 4. Other Exercise Information Content eTable 10. Format and Delivery of Exercise Information Communicated to Breast Cancer Survivors eTable 11. Intervention Outcomes and Effects eReferences. [file jamanetwopen-e258862-s001.pdf]

Supplemental Online Content

Wilson OWA, Wojcik KM, Jayasekera J, et al. Exercise communication for breast cancer survivors: a systematic scoping review. *JAMA Netw Open*. 2025;8(5):e258862. doi:10.1001/jamanetworkopen.2025.8862

eAppendix 1. Search Strategies

eMethods.

eTable 1. Inclusion and Exclusion Criteria for the Scoping Review

eTable 2. Information Extracted for This Scoping Review

eFigure 1. Article Identification Process Using PRISMA Research Framework

eAppendix 3. Study and Breast Cancer Survivor Characteristics

eTable 3. Participant Characteristics

eTable 4. Participant Demographic Characteristics

eTable 5. Participant Clinical Characteristics

eTable 6. Participant Contextual Characteristics

eTable 7. Intervention Characteristics and Nonexercise Communication Components

eTable 8. Content of Exercise Information Communicated to Breast Cancer Survivors

eTable 9. Why Exercise Information Was Communicated

eAppendix 4. Other Exercise Information Content

eTable 10. Format and Delivery of Exercise Information Communicated to Breast Cancer Survivors

eTable 11. Intervention Outcomes and Effects

eReferences.

This supplemental material has been provided by the authors to give readers additional information about their work.

© 2025 Wilson OWA et al. *JAMA Network Open*.

eAppendix 1. Search strategies

Note. Searches were conducted from database inception with no date limits

Database: PubMed/MEDLINE
Platform: National Library of Medicine
Date Searched: 4/17/2024

	Concept:	Search Strategy:
#1	Exercise	"Exercise"[Mesh] OR "Exercise Movement Techniques"[Mesh] OR "Exercise Therapy"[Mesh] OR "exercise*"[Title/Abstract] OR "Physical Fitness"[Mesh] OR "physical fitness"[Title/Abstract] OR "physical activit*"[Title/Abstract] OR "physical performanc*"[Title/Abstract] OR "Physical Exertion"[Mesh] OR "physical exertion"[Title/Abstract] OR "Physical Endurance"[Mesh] OR "physical endurance"[Title/Abstract:~4] OR "endurance training"[Title/Abstract] OR "physical intensit*"[Title/Abstract] OR "Recreation"[Mesh:NoExp] OR "recreational activit*"[Title/Abstract] OR "Sports"[Mesh] OR "sport*"[Title/Abstract] OR "stretching"[Title/Abstract] OR "Yoga"[Mesh] OR "yoga"[Title/Abstract] OR "Tai Ji"[Mesh] OR "Tai Ji"[Title/Abstract] OR "Tai Chi"[Title/Abstract] OR "muscle strength*"[Title/Abstract] OR "muscle strengthening"[Title/Abstract:~4] OR "resistance training"[Title/Abstract] OR "strength training"[Title/Abstract:~4] OR "muscle strength*"[Title/Abstract] OR "weight training"[Title/Abstract] OR "weightlifting"[Title/Abstract] OR "weight lifting"[Title/Abstract] OR "Walking"[Mesh] OR "walk*"[Title/Abstract] OR "gait"[Title/Abstract] OR "jog"[Title/Abstract] OR "jogging"[Title/Abstract] OR "running"[Title/Abstract] OR "swimming"[Title/Abstract] OR "aerobic*"[Title/Abstract] OR "exertion*"[Title/Abstract] OR "isometric*"[Title/Abstract] OR "energy expenditure*"[Title/Abstract] OR "energy balanc*"[Title/Abstract] OR "energy intake*"[Title/Abstract] OR "circuit*"[Title/Abstract] OR "high intensity interval*"[Title/Abstract] OR "high intensity intermittent*"[Title/Abstract] OR "HIIT"[Title/Abstract] OR "sprint interval*"[Title/Abstract] OR "stair climbing"[Title/Abstract:~4] OR "calisthenic*"[Title/Abstract] OR "cycling*"[Title/Abstract] OR "biking*"[Title/Abstract] OR "Sedentary Behavior"[Mesh] OR "sedentary"[Title/Abstract] OR "physical inactivit*"[Title/Abstract] OR "Dancing"[Mesh] OR "Dance Therapy"[Mesh] OR "dance*"[Title/Abstract] OR "dancing"[Title/Abstract] OR "pilate*"[Title/Abstract] OR "Pliability"[Mesh] OR "pliability"[Title/Abstract] OR "flexibilit*"[Title/Abstract] OR "Fitness Trackers"[Mesh] OR "fitness tracker*"[Title/Abstract] OR "smartwatch*"[Title/Abstract] OR "activity track*"[Title/Abstract]
#2	Cancer Survivor	"Cancer Survivors"[Mesh] OR "cancer survivor"[Title/Abstract:~4] OR "cancer survivors"[Title/Abstract:~4] OR "surviving cancer"[Title/Abstract:~2] OR "cancer survivorship*"[Title/Abstract] OR "cancer survivors"[Title/Abstract] OR "cancer diagnos*"[Title/Abstract] OR (("Survivors"[Mesh] OR "Survivorship"[Mesh] OR "surviv*"[Title/Abstract]) AND ("Neoplasms"[Mesh] OR "neoplas*"[Title/Abstract] OR "cancer*"[Title/Abstract] OR "carcinoma*"[Title/Abstract] OR "adenocarcinoma*"[Title/Abstract] OR "tumor*"[Title/Abstract] OR "tumour*"[Title/Abstract] OR "malignan*"[Title/Abstract] OR "melanoma*"[Title/Abstract] OR "sarcoma*"[Title/Abstract] OR "lymphoma*"[Title/Abstract] OR "Leukemia"[Mesh] OR "leukemia*"[Title/Abstract]))
#3	Communica-tion	"Communication"[Mesh:NoExp] OR "Health Communication"[Mesh] OR "communicat*"[Title/Abstract] OR "Access to Information"[Mesh] OR "Information Dissemination"[Mesh] OR "Information Seeking Behavior"[Mesh] OR "Information Literacy"[Mesh] OR "literacy"[Title/Abstract] OR "information"[Title/Abstract] OR "Communication Barriers"[Mesh] OR "Disinformation"[Mesh] OR "disinformation*"[Title/Abstract] OR "missinform*"[Title/Abstract] OR "Text Messaging"[Mesh] OR "messag*"[Title/Abstract] OR "Persuasive Communication"[Mesh] OR "persuas*"[Title/Abstract] OR "Marketing"[Mesh] OR "marketing*"[Title/Abstract] OR "Advertising"[Mesh] OR "advertis*"[Title/Abstract] OR "Internet"[Mesh:NoExp] OR "Internet*"[Title/Abstract] OR "Communications Media"[Mesh] OR "Social Media"[Mesh] OR "social media*"[Title/Abstract] OR "digital health*"[Title/Abstract] OR "electronic health*"[Title/Abstract] OR "Mass Media"[Mesh] OR "mass media*"[Title/Abstract] OR "television*"[Title/Abstract] OR "radio"[Title/Abstract] OR "movie*"[Title/Abstract] OR "social network*"[Title/Abstract] OR "Twitter*"[Title/Abstract] OR "Blogging"[Mesh] OR

© 2025 Wilson OWA et al. *JAMA Network Open*.

		"blog*"[Title/Abstract] OR "Facebook*"[Title/Abstract] OR "tiktok*"[Title/Abstract] OR "Youtube*"[Title/Abstract] OR "Instagram*"[Title/Abstract] OR "texting*"[Title/Abstract] OR "Consumer Health Information"[Mesh] OR "consumer health"[Title/Abstract] OR_"face to face"[Title/Abstract] OR "center based"[Title/Abstract] OR "Mobile Applications"[Mesh] OR "mobile app*"[Title/Abstract] OR "portal*"[Title/Abstract] OR "email*"[Title/Abstract] OR "mail*"[Title/Abstract] OR "ehealth*"[Title/Abstract] OR "e-health*"[Title/Abstract] OR "mhealth*"[Title/Abstract] OR "m-health*"[Title/Abstract] OR "telecommunication*"[Title/Abstract] OR "teleconferenc*"[Title/Abstract] OR "video conferenc*"[Title/Abstract]
#4		#1 AND #2 AND #3
#5	Limits & Filters	((#1 AND #2 AND #3) NOT ("Animals"[Mesh] NOT ("Animals"[Mesh] AND "Humans"[Mesh]))) NOT ("Letter"[Publication Type] OR "Editorial" [Publication Type] OR "Comment"[Publication Type] OR "News"[Publication Type] OR "Retracted Publication"[Publication Type] OR "Retraction of Publication"[Publication Type] OR "retraction of publication"[Title/Abstract] OR "retraction notice"[Title] OR "retracted publication"[Title/Abstract] OR "protocol*"[Title] OR "symposium*"[Title/Abstract] OR "Congress"[Publication Type] OR "Consensus Development Conference"[Publication Type] OR "conference*"[Title] OR "conference abstract*"[Title/Abstract] OR "conference proceeding*"[Title/Abstract] OR "conference paper*"[Title/Abstract] OR "conference review*"[Title/Abstract] OR "proceeding*"[Title]) AND Filters: English

Database: Cochrane Library
Platform: Wiley & Sons
Date Searched: 4/17/2024

	Concept:	Search Strategy:
#1	Exercise	([mh "Exercise"] OR [mh "Exercise Movement Techniques"] OR [mh "Exercise Therapy"] OR exercise* OR [mh "Physical Fitness"] OR "physical fitness" OR (physical NEXT activit*) OR (physical NEXT performanc*) OR [mh "Physical Exertion"] OR "physical exertion" OR [mh "Physical Endurance"] OR (physical NEAR/4 endurance) OR "endurance training" OR (physical NEXT intensit*) OR [mh ^"Recreation"] OR (recreational NEXT activit*) OR [mh "Sports"] OR sport* OR stretching OR [mh "Yoga"] OR yoga OR [mh "Tai Ji"] OR "Tai Ji" OR "Tai Chi" OR (muscle NEXT strength*) OR (muscle NEAR/4 strengthening) OR "resistance training" OR (strength NEAR/4 training) OR (muscle NEXT strength*) OR "weight training" OR weightlifting OR "weight lifting" OR [mh "Walking"] OR walk* OR gait OR jog OR jogging OR running OR swimming OR aerobic* OR exertion* OR isometric* OR (energy NEXT expenditure*) OR (energy NEXT balanc*) OR (energy NEXT intake*) OR circuit* OR "high intensity interval" OR "high intensity intervals" OR "high intensity intermittent" OR "high intensity intermittents" OR "HIIT" OR (sprint NEXT interval*) OR (stair NEAR/4 climbing) OR calisthenic* OR cycling* OR biking* OR [mh "Sedentary Behavior"] OR sedentary OR (physical NEXT inactivit*) OR [mh "Dancing"] OR [mh "Dance Therapy"] OR dance* OR dancing OR pilate* OR [mh "Pliability"] OR pliability OR flexibilit* OR [mh "Fitness Trackers"] OR (fitness NEXT tracker*) OR smartwatch* OR (activity NEXT track*)):ti,ab,kw
#2	Cancer Survivor	([mh "Cancer Survivors"] OR (cancer* NEAR/4 survivor*) OR (surviving NEAR/2 cancer*) OR (cancer NEXT survivorship*) OR (cancer NEXT diagnos*) OR (([mh "Survivors"] OR [mh "Survivorship"] OR surviv*) AND ([mh "Neoplasms"] OR neoplas* OR cancer* OR carcinoma* OR adenocarcinoma* OR tumor* OR tumour* OR malignan* OR melanoma* OR sarcoma* OR lymphoma* OR [mh "Leukemia"] OR leukemia*))):ti,ab,kw

© 2025 Wilson OWA et al. *JAMA Network Open*.

#3	Communication	([mh ^"Communication"] OR [mh "Health Communication"] OR communicat* OR [mh "Access to Information"] OR [mh "Information Dissemination"] OR [mh "Information Seeking Behavior"] OR [mh "Information Literacy"] OR literacy OR information OR [mh "Communication Barriers"] OR [mh "Disinformation"] OR disinformation* OR missinform* OR [mh "Text Messaging"] OR messag* OR [mh "Persuasive Communication"] OR persuas* OR [mh "Marketing"] OR marketing* OR [mh "Advertising"] OR advertis* OR [mh ^"Internet"] OR Internet* OR [mh "Communications Media"] OR [mh "Social Media"] OR (social NEXT media*) OR (digital NEXT health*) OR (electronic NEXT health*) OR [mh "Mass Media"] OR (mass NEXT media*) OR television* OR radio OR movie* OR (social NEXT network*) OR Twitter* OR [mh "Blogging"] OR blog* OR Facebook* OR tiktok* OR Youtube* OR Instagram* OR texting* OR [mh "Consumer Health Information"] OR "consumer health" OR "face to face" OR "center based" OR [mh "Mobile Applications"] OR (mobile NEXT app*) OR portal* OR email* OR mail* OR ehealth* OR e-health* OR mhealth* OR m-health* OR telecommunication* OR teleconferenc* OR (video NEXT conferenc*)):ti,ab,kw
#4		#1 AND #2 AND #3 (Word variations have been searched)
#5	Limits	[mh "Letter"] OR [mh "Editorial"] OR [mh "Comment"] OR [mh "News"] OR [mh "Retracted Publication"] OR [mh "Retraction of Publication"] OR "retraction of publication" OR "retracted publication" OR symposium* OR [mh "Congress"] OR [mh "Consensus Development Conference"] OR (conference abstract*) OR (conference NEXT proceeding*) OR (conference NEXT paper*) OR (conference NEXT review*)"
#6	Limits	(protocol* OR conference* OR "retraction notice" OR proceeding*):ti
#7		#5 OR #6 (Word variations have been searched)
#8		#4 NOT #7 (Word variations have been searched)

Database: Embase
Platform: Elsevier
Date Searched: 4/17/2024

	Concept:	Search Strategy:
#1	Exercise	'exercise'/exp OR 'kinesiotherapy'/exp OR 'fitness'/exp OR 'endurance'/exp OR 'physical performance'/exp OR 'physical activity, capacity and performance'/de OR 'recreation'/exp OR 'recreation'/de OR 'dancing'/de OR 'dancing'/exp OR 'sport'/exp OR 'yoga'/exp OR 'tai chi'/exp OR 'walking'/exp OR 'sedentary lifestyle'/exp OR 'dance therapy'/exp OR 'pliability'/exp OR 'activity tracker'/exp OR 'exercise*':ab,ti OR 'physical fitness':ab,ti OR 'physical activit*':ab,ti OR 'physical performanc*':ab,ti OR 'physical exertion':ab,ti OR ((physical NEAR/4 endurance*):ab,ti) OR 'endurance training':ab,ti OR 'physical intensit*':ab,ti OR 'recreational activit*':ab,ti OR 'sport*':ab,ti OR 'stretching':ab,ti OR 'yoga':ab,ti OR 'tai ji':ab,ti OR 'tai chi':ab,ti OR ((muscle NEAR/4 strengthening):ab,ti) OR 'resistance training':ab,ti OR ((strength NEAR/4 training):ab,ti) OR 'muscle strength*':ab,ti OR 'weight training':ab,ti OR 'weightlifting':ab,ti OR 'weight lifting':ab,ti OR 'walk*':ab,ti OR 'gait':ab,ti OR 'jog':ab,ti OR 'jogging':ab,ti OR 'running':ab,ti OR 'swimming':ab,ti OR 'aerobic*':ab,ti OR 'exertion*':ab,ti OR 'isometric*':ab,ti OR 'energy expenditure*':ab,ti OR 'energy balanc*':ab,ti OR 'energy intake*':ab,ti OR 'circuit*':ab,ti OR 'high intensity interval*':ab,ti OR 'high intensity intermittent*':ab,ti OR 'hiit':ab,ti OR 'sprint interval*':ab,ti OR ((stair NEAR/4 climbing):ab,ti) OR 'calisthenic*':ab,ti OR 'cycling*':ab,ti OR 'biking*':ab,ti OR 'sedentary':ab,ti OR 'physical inactivit*':ab,ti OR 'dance*':ab,ti OR 'dancing':ab,ti OR 'pilate*':ab,ti OR 'pliability':ab,ti OR 'flexibilit*':ab,ti OR 'fitness tracker*':ab,ti OR 'smartwatch*':ab,ti OR 'activity track*':ab,ti
#2	Cancer Survivor	'cancer survival'/exp OR 'cancer survivor'/exp OR ((cancer NEAR/4 survivor*):ab,ti) OR ((cancer NEAR/4 survivors):ab,ti) OR ((surviving NEAR/2 cancer*):ab,ti) OR 'cancer survivorship*':ab,ti OR 'cancer survivors':ab,ti OR 'cancer diagnos*':ab,ti OR ('surviv*':ab,ti AND ('neoplas*':ab,ti OR 'cancer*':ab,ti OR 'carcinoma*':ab,ti OR 'adenocarcinoma*':ab,ti OR 'tumor*':ab,ti OR 'tumour*':ab,ti OR 'malignan*':ab,ti OR 'melanoma*':ab,ti OR 'sarcoma*':ab,ti OR 'lymphoma*':ab,ti OR 'leukemia*':ab,ti))

© 2025 Wilson OWA et al. *JAMA Network Open*.

#3	Communication	'interpersonal communication'/de OR 'medical information'/exp OR 'access to information'/exp OR 'information dissemination'/exp OR 'information seeking'/exp OR 'information literacy'/exp OR 'communication barrier'/exp OR 'disinformation'/exp OR 'text messaging'/exp OR 'persuasive communication'/exp OR 'marketing'/exp OR 'advertising'/exp OR 'internet'/de OR 'mass medium'/de OR 'social media'/de OR 'blogging'/de OR 'e-mail'/de OR 'radio'/de OR 'television'/de OR 'telecommunication'/de OR 'videoconferencing'/de OR 'web conferencing'/de OR 'consumer health information'/exp OR 'mobile application'/de OR 'communicat*':ab,ti OR 'literacy':ab,ti OR 'information':ab,ti OR 'disinformation*':ab,ti OR 'missinform*':ab,ti OR 'messag*':ab,ti OR 'persuas*':ab,ti OR 'marketing*':ab,ti OR 'advertis*':ab,ti OR 'internet*':ab,ti OR 'social media*':ab,ti OR 'digital health*':ab,ti OR 'electronic health*':ab,ti OR 'mass media*':ab,ti OR 'television*':ab,ti OR 'radio':ab,ti OR 'movie*':ab,ti OR 'social network*':ab,ti OR 'twitter*':ab,ti OR 'blog*':ab,ti OR 'facebook*':ab,ti OR 'tiktok*':ab,ti OR 'youtube*':ab,ti OR 'instagram*':ab,ti OR 'texting*':ab,ti OR 'consumer health':ab,ti OR 'face to face':ab,ti OR 'center based':ab,ti OR 'mobile app*':ab,ti OR 'portal*':ab,ti OR 'email*':ab,ti OR 'mail*':ab,ti OR 'ehealth*':ab,ti OR 'e-health*':ab,ti OR 'mhealth*':ab,ti OR 'm-health*':ab,ti OR 'telecommunication*':ab,ti OR 'teleconferenc*':ab,ti OR 'video conferenc*':ab,ti
#4		#1 AND #2 AND #3
#5	Limits & Filters	#1 AND #2 AND #3 AND ([article]/lim OR [article in press]/lim) AND [english]/lim NOT ([animals]/lim NOT ([animals]/lim AND [humans]/lim)) NOT ('editorial'/exp OR 'letter'/exp OR 'note'/exp OR 'abstract report'/exp OR 'conference paper'/exp OR 'review'/exp OR 'systematic review'/exp OR 'systematic review (topic)'/exp OR 'meta analysis'/exp OR 'meta analysis (topic)'/exp OR 'case report'/exp OR 'practice guideline'/exp OR 'retraction notice'/exp OR 'retraction of publication':ab,ti OR 'retraction notice':ti OR 'retracted publication':ab,ti OR 'systematic review':ab,ti OR 'meta analysis':ab,ti OR 'meta analyses':ab,ti OR 'review':ti OR 'protocol':ti OR 'protocols':ti OR 'guideline*':ti OR 'case report*':ab,ti OR [conference abstract]/lim OR [conference paper]/lim OR [conference review]/lim OR 'symposium*':ab,ti OR 'conference abstract*':ab,ti OR 'conference review*':ab,ti OR 'conference proceeding*':ab,ti OR 'conference paper*':ab,ti)

Database: Web of Science (Core Collection)
Platform: Clarivate Analytics
Date Searched: 4/17/2024

	Concept:	Search Strategy:
#1	Exercise	(TI=("exercise*" OR "physical fitness" OR "physical activit*" OR "physical performanc*" OR "physical exertion" OR (physical NEAR/4 endurance*) OR "endurance training" OR "physical intensit*" OR "recreational activit*" OR "sport*" OR "stretching" OR "yoga" OR "Tai Ji" OR "Tai Chi" OR "muscle strength*" OR (muscle NEAR/4 strengthening) OR "resistance training" OR (strength NEAR/4 training) OR "muscle strength*" OR "weight training" OR "weightlifting" OR "weight lifting" OR "walk*" OR "gait" OR "jog" OR "jogging" OR "running" OR "swimming" OR "aerobic*" OR "exertion*" OR "isometric*" OR "energy expenditure*" OR "energy balanc*" OR "energy intake*" OR "circuit*" OR "high intensity interval*" OR "high intensity intermittent*" OR "HIIT" OR "sprint interval*" OR (stair NEAR/4 climbing) OR "calisthenic*" OR "cycling*" OR "biking*" OR "sedentary" OR "physical inactivit*" OR "dance*" OR "dancing" OR "pilate*" OR "pliability" OR "flexibilit*" OR "fitness tracker*" OR "smartwatch*" OR "activity track*")) OR AB=("exercise*" OR "physical fitness" OR "physical activit*" OR "physical performanc*" OR "physical exertion" OR (physical NEAR/4 endurance*) OR "endurance training" OR "physical intensit*" OR "recreational activit*" OR "sport*" OR "stretching" OR "yoga" OR "Tai Ji" OR "Tai Chi" OR "muscle strength*" OR (muscle NEAR/4 strengthening) OR "resistance training" OR (strength NEAR/4 training) OR "muscle strength*" OR "weight training" OR "weightlifting" OR "weight lifting" OR "walk*" OR "gait" OR "jog" OR "jogging" OR "running" OR "swimming" OR "aerobic*" OR "exertion*" OR "isometric*" OR "energy expenditure*" OR "energy balanc*" OR "energy intake*" OR "circuit*" OR "high intensity interval*" OR "high intensity intermittent*" OR "HIIT" OR "sprint interval*" OR (stair NEAR/4 climbing) OR "calisthenic*" OR "cycling*" OR "biking*" OR "sedentary" OR "physical inactivit*" OR "dance*" OR "dancing" OR "pilate*" OR "pliability" OR "flexibilit*" OR "fitness tracker*" OR "smartwatch*" OR "activity track*")
#2	Cancer Survivor	(TI=("cancer survivorship*" OR "cancer diagnos*" OR (cancer NEAR/4 survivor*) OR (cancer NEAR/4 survivors) OR (surviving NEAR/2 cancer*) OR ("surviv*" AND ("neoplas*" OR "cancer*" OR "carcinoma*" OR "adenocarcinoma*" OR "tumor*" OR "tumour*" OR "malignan*" OR "melanoma*" OR "sarcoma*" OR "lymphoma*" OR "leukemia*")))) OR AB=("cancer

© 2025 Wilson OWA et al. *JAMA Network Open*.

		survivorship*" OR "cancer diagnos*" OR (cancer NEAR/4 survivor*) OR (cancer NEAR/4 survivors) OR (surviving NEAR/2 cancer*) OR ("surviv*" AND ("neoplas*" OR "cancer*" OR "carcinoma*" OR "adenocarcinoma*" OR "tumor*" OR "tumour*" OR "malignan*" OR "melanoma*" OR "sarcoma*" OR "lymphoma*" OR "leukemia*")))
#3	Communication	(TI=("communicat*" OR "literacy" OR "information" OR "disinformation*" OR "missinform*" OR "messag*" OR "persuas*" OR "marketing*" OR "advertis*" OR "Internet*" OR "social media*" OR "digital health*" OR "electronic health*" OR "mass media*" OR "television*" OR "radio" OR "movie*" OR "social network*" OR "Twitter*" OR "blog*" OR "Facebook*" OR "tiktok*" OR "Youtube*" OR "Instagram*" OR "texting*" OR "consumer health" OR "face to face" OR "center based" OR "mobile app*" OR "portal*" OR "email*" OR "mail*" OR "ehealth*" OR "e-health*" OR "mhealth*" OR "m-health*" OR "telecommunication*" OR "teleconferenc*" OR "video conferenc*")) OR AB=("communicat*" OR "literacy" OR "information" OR "disinformation*" OR "missinform*" OR "messag*" OR "persuas*" OR "marketing*" OR "advertis*" OR "Internet*" OR "social media*" OR "digital health*" OR "electronic health*" OR "mass media*" OR "television*" OR "radio" OR "movie*" OR "social network*" OR "Twitter*" OR "blog*" OR "Facebook*" OR "tiktok*" OR "Youtube*" OR "Instagram*" OR "texting*" OR "consumer health" OR "face to face" OR "center based" OR "mobile app*" OR "portal*" OR "email*" OR "mail*" OR "ehealth*" OR "e-health*" OR "mhealth*" OR "m-health*" OR "telecommunication*" OR "teleconferenc*" OR "video conferenc*")
#4		#1 AND #2 AND #3
#5	Limits & Filters	#1 AND #2 AND #3 and Mice or Animals (Exclude – MeSH Headings) and Preprint Citation Index (Exclude – Database) and Meeting or Abstract or Book or Editorial Material or Letter or Retracted Publication (Exclude – Document Types) and English (Languages) and Web of Science Core Collection (Database)

Database: Communication and Mass Media Complete
Platform: EBSCO*host*
Date Searched: 4/17/2024

	Concept:	Search Strategy:
#S1	Exercise	(DE "MASS media & sports" OR DE "RADIO broadcasting of sports" OR DE "SPORTS journalism" OR DE "TELEVISED sports" OR DE "DANCE" OR DE "PHYSICAL fitness mobile apps") OR TI ("exercise*" OR "physical fitness" OR "physical activit*" OR "physical performanc*" OR "physical exertion" OR (physical N4 endurance*") OR "endurance training" OR "physical intensit*" OR "recreational activit*" OR "sport*" OR "stretching" OR "yoga" OR "Tai Ji" OR "Tai Chi" OR "muscle strength*" OR (muscle N4 strengthening) OR "resistance training" OR (strength N4 training) OR "muscle strength*" OR "weight training" OR "weightlifting" OR "weight lifting" OR "walk*" OR "gait" OR "jog" OR "jogging" OR "running" OR "swimming" OR "aerobic*" OR "exertion*" OR "isometric*" OR "energy expenditure*" OR "energy balanc*" OR "energy intake*" OR "circuit*" OR "high intensity interval*" OR "high intensity intermittent*" OR "HIIT" OR "sprint interval*" OR (stair N4 climbing) OR "calisthenic*" OR "cycling*" OR "biking*" OR "sedentary" OR "physical inactivit*" OR "dance*" OR "dancing" OR "pilate*" OR "pliability" OR "flexibilit*" OR "fitness tracker*" OR "smartwatch*" OR "activity track*") OR AB ("exercise*" OR "physical fitness" OR "physical activit*" OR "physical performanc*" OR "physical exertion" OR (physical N4 endurance*") OR "endurance training" OR "physical intensit*" OR "recreational activit*" OR "sport*" OR "stretching" OR "yoga" OR "Tai Ji" OR "Tai Chi" OR "muscle strength*" OR (muscle N4 strengthening) OR "resistance training" OR (strength N4 training) OR "muscle strength*" OR "weight training" OR "weightlifting" OR "weight lifting" OR "walk*" OR "gait" OR "jog" OR "jogging" OR "running" OR "swimming" OR "aerobic*" OR "exertion*" OR "isometric*" OR "energy expenditure*" OR "energy balanc*" OR "energy intake*" OR "circuit*" OR "high intensity interval*" OR "high intensity intermittent*" OR "HIIT" OR "sprint interval*" OR (stair N4 climbing) OR "calisthenic*" OR "cycling*" OR "biking*" OR "sedentary" OR "physical inactivit*" OR "dance*" OR "dancing" OR "pilate*" OR "pliability" OR "flexibilit*" OR "fitness tracker*" OR "smartwatch*" OR "activity track*")
#S2	Cancer Survivor	"cancer survivorship*" OR "cancer diagnos*" OR (cancer N4 surviv*) OR ("surviv*" AND ("neoplas*" OR "cancer*" OR "carcinoma*" OR "adenocarcinoma*" OR "tumor*" OR "tumour*" OR "malignan*" OR "melanoma*" OR "sarcoma*" OR "lymphoma*" OR "leukemia*"))

© 2025 Wilson OWA et al. *JAMA Network Open*.

#S3	Communication	(DE "COMMUNICATION") OR (DE "MEDICAL communication") OR (DE "ACCESS to information") OR (DE "INFORMATION dissemination") OR (DE "MASS media")) OR (DE "DIGITAL literacy")) OR (DE "SOCIAL media")) OR (DE "COMMUNICATION barriers")) OR (DE "DISINFORMATION")) OR (DE "TEXT messages")) OR (DE "PERSUASION (Rhetoric)")) OR (DE "MARKETING")) OR (DE "ADVERTISING")) OR (DE "INTERNET")) OR (DE "MASS media")) OR (DE "SOCIAL media")) OR (DE "BLOGS")) OR (DE "MOBILE apps")) OR TI ("communicat*" OR "literacy" OR "information" OR "disinformation*" OR "missinform*" OR "messag*" OR "persuas*" OR "marketing*" OR "advertis*" OR "Internet*" OR "social media*" OR "digital health*" OR "electronic health*" OR "mass media*" OR "television*" OR "radio" OR "movie*" OR "social network*" OR "Twitter*" OR "blog*" OR "Facebook*" OR "tiktok*" OR "Youtube*" OR "Instagram*" OR "texting*" OR "consumer health" OR "face to face" OR "center based" OR "mobile app*" OR "portal*" OR "email*" OR "mail*" OR "ehealth*" OR "e-health*" OR "mhealth*" OR "m-health*" OR "telecommunication*" OR "teleconferenc*" OR "video conferenc*") OR AB ("communicat*" OR "literacy" OR "information" OR "disinformation*" OR "missinform*" OR "messag*" OR "persuas*" OR "marketing*" OR "advertis*" OR "Internet*" OR "social media*" OR "digital health*" OR "electronic health*" OR "mass media*" OR "television*" OR "radio" OR "movie*" OR "social network*" OR "Twitter*" OR "blog*" OR "Facebook*" OR "tiktok*" OR "Youtube*" OR "Instagram*" OR "texting*" OR "consumer health" OR "face to face" OR "center based" OR "mobile app*" OR "portal*" OR "email*" OR "mail*" OR "ehealth*" OR "e-health*" OR "mhealth*" OR "m-health*" OR "telecommunication*" OR "teleconferenc*" OR "video conferenc*")
#4		#S1 AND #S2 AND #S3
#5	Limits & Filters	((#S1 AND #S2 AND #S3) Expanders - Apply equivalent subjects<br>Narrow by Language: - English Search modes - Boolean/Phrase

Database: PsycInfo
Platform: American Psychological Association
Date Searched: 4/17/2024

	Concept:	Search Strategy:
	Exercise AND Cancer Survivor AND Communication	((IndexTermsFilt: ("Exercise") OR IndexTermsFilt: ("Exercise Therapy") OR IndexTermsFilt: ("Sports") OR IndexTermsFilt: ("Recreation") OR IndexTermsFilt: ("Walking") OR IndexTermsFilt: ("Yoga") OR IndexTermsFilt: ("Sedentary Behavior") OR IndexTermsFilt: ("Dance Therapy")) OR title: ("exercise*" OR "physical fitness" OR "physical activit*" OR "physical performanc*" OR "physical exertion" OR (physical NEAR/4 endurance* ") OR " endurance training " OR " physical intensit* " OR " recreational activit* " OR " sport* " OR " stretching " OR " yoga " OR " Tai Ji " OR " Tai Chi " OR " muscle strength* " OR (muscle NEAR/4 strengthening) OR " resistance training " OR (strength NEAR/4 training) OR " muscle strength* " OR " weight training " OR " weightlifting " OR " weight lifting " OR " walk* " OR " gait " OR " jog " OR " jogging " OR " running " OR " swimming " OR " aerobic* " OR " exertion* " OR " isometric* " OR " energy expenditure* " OR " energy balanc* " OR " energy intake* " OR " circuit* " OR " high intensity interval* " OR " high intensity intermittent* " OR " HIIT " OR " sprint interval* " OR (stair NEAR/4 climbing) OR " calisthenic* " OR " cycling* " OR " biking* " OR " sedentary " OR " physical inactivit* " OR " dance* " OR " dancing " OR " pilate* " OR " pliability " OR " flexibilit* " OR " fitness tracker* " OR " smartwatch* " OR " activity track* ") OR Abstract:(" exercise* " OR " physical fitness " OR " physical activit* " OR " physical performanc* " OR " physical exertion " OR (physical NEAR/4 endurance*") OR "endurance training" OR "physical intensit*" OR "recreational activit*" OR "sport*" OR "stretching" OR "yoga" OR "Tai Ji" OR "Tai Chi" OR "muscle strength*" OR (muscle NEAR/4 strengthening) OR "resistance training" OR (strength NEAR/4 training) OR "muscle strength*" OR "weight training" OR "weightlifting" OR "weight lifting" OR "walk*" OR "gait" OR "jog" OR "jogging" OR "running" OR "swimming" OR "aerobic*" OR "exertion*" OR "isometric*" OR "energy expenditure*" OR "energy balanc*" OR "energy intake*" OR "circuit*" OR "high intensity interval*" OR "high intensity intermittent*" OR "HIIT" OR "sprint interval*" OR (stair NEAR/4 climbing) OR "calisthenic*" OR "cycling*" OR "biking*" OR "sedentary" OR "physical inactivit*" OR "dance*" OR "dancing" OR "pilate*" OR "pliability" OR "flexibilit*" OR "fitness tracker*" OR "smartwatch*" OR "activity track*")) AND ((IndexTermsFilt: ("Neoplasms")

© 2025 Wilson OWA et al. *JAMA Network Open*.

		AND IndexTermsFilt: ("Survivors")) OR (title: (cancer NEAR/4 survivor*)) OR (title: (cancer NEAR/4 survivors)) OR (title: (surviving NEAR/2 cancer*)) OR title: ("cancer survivorship*") OR title: ("cancer survivors") OR title: ("cancer diagnos*") OR (abstract: (cancer NEAR/4 survivor*)) OR (abstract: (cancer NEAR/4 survivors)) OR (abstract: (surviving NEAR/2 cancer*)) OR abstract: ("cancer survivorship*") OR abstract: ("cancer survivors") OR abstract: ("cancer diagnos*")) AND ((IndexTermsFilt: ("Communication") OR IndexTermsFilt: ("Information Dissemination") OR IndexTermsFilt: ("Information Seeking") OR IndexTermsFilt: ("Information Literacy") OR IndexTermsFilt: ("Text Messaging") OR IndexTermsFilt: ("Marketing") OR IndexTermsFilt: ("Advertising") OR IndexTermsFilt: ("Digital Interventions") OR IndexTermsFilt: ("Online Experiments") OR IndexTermsFilt: ("Communications Media") OR IndexTermsFilt: ("Social Media") OR IndexTermsFilt: ("Mass Media") OR IndexTermsFilt: ("Blog") OR IndexTermsFilt: ("Mobile Applications")) OR (title: ("communicat*") OR title: ("literacy") OR title: ("information") OR title: ("disinformation*") OR title: ("missinform*") OR title: ("messag*") OR title: ("persuas*") OR title: ("marketing*") OR title: ("advertis*") OR title: ("Internet*") OR title: ("social media*") OR title: ("digital health*") OR title: ("electronic health*") OR title: ("mass media*") OR title: ("television*") OR title: ("radio") OR title: ("movie*") OR title: ("social network*") OR title: ("Twitter*") OR title: ("blog*") OR title: ("Facebook*") OR title: ("tiktok*") OR title: ("Youtube*") OR title: ("Instagram*") OR title: ("texting*") OR title: ("consumer health") OR title: ("face to face") OR title: ("center based") OR title: ("mobile app*") OR title: ("portal*") OR title: ("email*") OR title: ("mail*") OR title: ("ehealth*") OR title: ("e-health*") OR title: ("mhealth*") OR title: ("m-health*") OR title: ("telecommunication*") OR title: ("teleconferenc*") OR title: ("video conferenc*")) OR (abstract: ("communicat*") OR abstract: ("literacy") OR abstract: ("information") OR abstract: ("disinformation*") OR abstract: ("missinform*") OR abstract: ("messag*") OR abstract: ("persuas*") OR abstract: ("marketing*") OR abstract: ("advertis*") OR abstract: ("Internet*") OR abstract: ("social media*") OR abstract: ("digital health*") OR abstract: ("electronic health*") OR abstract: ("mass media*") OR abstract: ("television*") OR abstract: ("radio") OR abstract: ("movie*") OR abstract: ("social network*") OR abstract: ("Twitter*") OR abstract: ("blog*") OR abstract: ("Facebook*") OR abstract: ("tiktok*") OR abstract: ("Youtube*") OR abstract: ("Instagram*") OR abstract: ("texting*") OR abstract: ("consumer health") OR abstract: ("face to face") OR abstract: ("center based") OR abstract: ("mobile app*") OR abstract: ("portal*") OR abstract: ("email*") OR abstract: ("mail*") OR abstract: ("ehealth*") OR abstract: ("e-health*") OR abstract: ("mhealth*") OR abstract: ("m-health*") OR abstract: ("telecommunication*") OR abstract: ("teleconferenc*") OR abstract: ("video conferenc*"))) AND Language: English AND Population Group: Human NOT Population Group: Animal NOT Document Type: Abstract Collection OR Chapter OR Column/Opinion OR Comment/Reply OR Dissertation OR Editorial OR Letter OR Retraction OR Review-Book AND Peer-Reviewed Journals only

© 2025 Wilson OWA et al. *JAMA Network Open*.

eMethods.

Protocol and Registration

This review was conducted in accordance with the Preferred Reporting Items for Systematic reviews and Meta-Analyses extension for Scoping Reviews (PRISMA-ScR) checklist.[2] The methodological framework proposed by Arksey and O'Malley, Levac and colleagues, and the Joanna Briggs Institute was followed.[3,4] This review was registered in Open Science Framework (doi: 10.17605/OSF.IO/YXV6W*)*.[5]

Eligibility Criteria

The focus of this review was studies describing the development and/or delivery of exercise communication for female breast cancer survivors (i.e., women diagnosed with breast cancer regardless of treatment status) in any geographical location or setting. It includes articles examining one-on-one communication, which were excluded from a previous review of physical activity messaging that screened articles at the title level and focused on the overall population (vs. breast cancer survivors only).[6] Articles that focused on wider communication techniques, such as public health messages, or only implied communication of exercise information were excluded. The search was limited to full-text articles written in English. There were no inclusion/exclusion criteria regarding sample size or the date of publication. Inclusion and exclusion criteria are detailed in eTable 1.

Information Sources and Search Strategy

The comprehensive search strategy of six databases (i.e., PubMed, PsycInfo, Embase, Scopus, Web of Science, and Cochrane Database of Systematic Reviews) included keywords, synonyms, Medical Subject Headings (MeSH), and Emtree terms related to physical activity, cancer survivorship, and counseling, messaging, or communication (Appendix 1). A librarian from the National Institutes of Health Library (GB) pilot tested 50 articles, and the search strategy was refined based on initial results. We conducted a comprehensive search of the literature from the inception of each database until April 2024. Reference lists of included articles were searched for relevant studies.

© 2025 Wilson OWA et al. *JAMA Network Open*.

Selection of Sources of Evidence

Covidence (Veritas Health Innovation, Melbourne, Australia) was used to complete the data screening process.[7] Titles and abstracts for all articles retrieved were screened for eligibility by two authors (OW, KW) based on the inclusion/exclusion criteria. Using the same criteria, articles were then reviewed at full text. Disagreements were resolved via discussion.

Data Charting Process and Items

Information was extracted on the following: study, survivor; and intervention characteristics (including duration, theoretical framework, and other relevant components); exercise communication content, format and delivery; and, intervention outcomes/effects. Information extracted on exercise communication content, format, and delivery were informed by the PAMC.[8] Details on extracted information are reported in eTable 2. Study authors were contacted via email for additional information and clarification if information was missing or unclear. Findings were synthesized and articles from single studies were combined to report findings at the study level.[9]

Synthesis of Results

Extracted information was summarized using a narrative approach, as outlined by Cochrane.[10] Additional information is available in Tables 1-5 and eTables 3-11. Heterogeneity in intervention design (e.g., dietary advice or fitness trackers in addition to exercise information) and evaluation (e.g., intervention length and outcomes) limited our ability to conduct a quantitative synthesis of intervention effects across all studies. Therefore, a narrative approach was used to summarize information. Since narrative approaches to synthesizing results based on statistical significance are limited,[10] data were interpreted based on an evaluation of the magnitude, direction, and precision of the effect estimates rather than binary significance testing.[11] This allowed us to evaluate the clinical relevance of the results where we categorized changes in outcomes as favorable, no change, or unfavorable. We stratified results into studies conducted within the U.S. and outside of the U.S. to facilitate further comparisons for study design and exercise communication content, format, and delivery.

© 2025 Wilson OWA et al. *JAMA Network Open*.

eTable 1. Inclusion and Exclusion Criteria for the Scoping Review

Criteria	Inclusion	Exclusion
Population	• Female breast cancer survivors	• Non-human subjects<br>• Non-females<br>• Non-breast cancer survivors
Concept	• Report details on the development and/or delivery of exercise communication	• Do not report details on the development and/or delivery of exercise communication
Context	• All countries, all contexts	• Not applicable
Study type	• Primary empirical research studies (e.g., randomized controlled trials, interventions and cross-sectional studies) available in full text	• Reviews (e.g., meta-analysis, systematic reviews, scoping reviews, narrative reviews)<br>• Editorials (e.g., perspectives, commentaries)<br>• Abstracts, Conference proceedings or posters<br>• Dissertations/theses<br>• Research protocols<br>• Case reports<br>• Patents<br>• Articles for full text cannot be obtained
Language	• English	• Not available in English

© 2025 Wilson OWA et al. *JAMA Network Open*.

eTable 2. Information Extracted for This Scoping Review

Domain	Information extracted
Study characteristics	• Study name and location (country, region)<br>• Study design<br>• Year of publication
Survivor characteristics	• Demographic (age, race and ethnicity, weight status, menopausal status)<br>• Clinical (stage at diagnosis, treatment (e.g., chemotherapy,), time since diagnosis)<br>• Contextual (education, income, employment, marital status)
Intervention characteristics	• Intervention and follow-up length<br>• Theoretical framework<br>• Additional exercise intervention component(s) (e.g. exercise trackers,)Non-exercise communication component(s) (e.g. diet)
Exercise communication content	• What exercise to do, such as frequency, intensity, duration of aerobic and muscle-strengthening exercise<br>• Why, or why not, to exercise (i.e., exercise benefits or risks)<br>• Communication individualization (i.e., tailoring)<br>• Source that content was based on (e.g., specific exercise guidelines)
Exercise communication format and delivery	• Written vs. verbal, including specific method (e.g., handouts vs. , face-to-face (including setting))<br>• Who delivered information (e.g. oncologists vs. cancer exercise specialists)
Intervention outcomes/effects	The effect of the communication intervention on behavioral (e.g. exercise), psychological (e.g., intention to exercise), and health (e.g., weight, quality of life) outcomes

© 2025 Wilson OWA et al. *JAMA Network Open*.

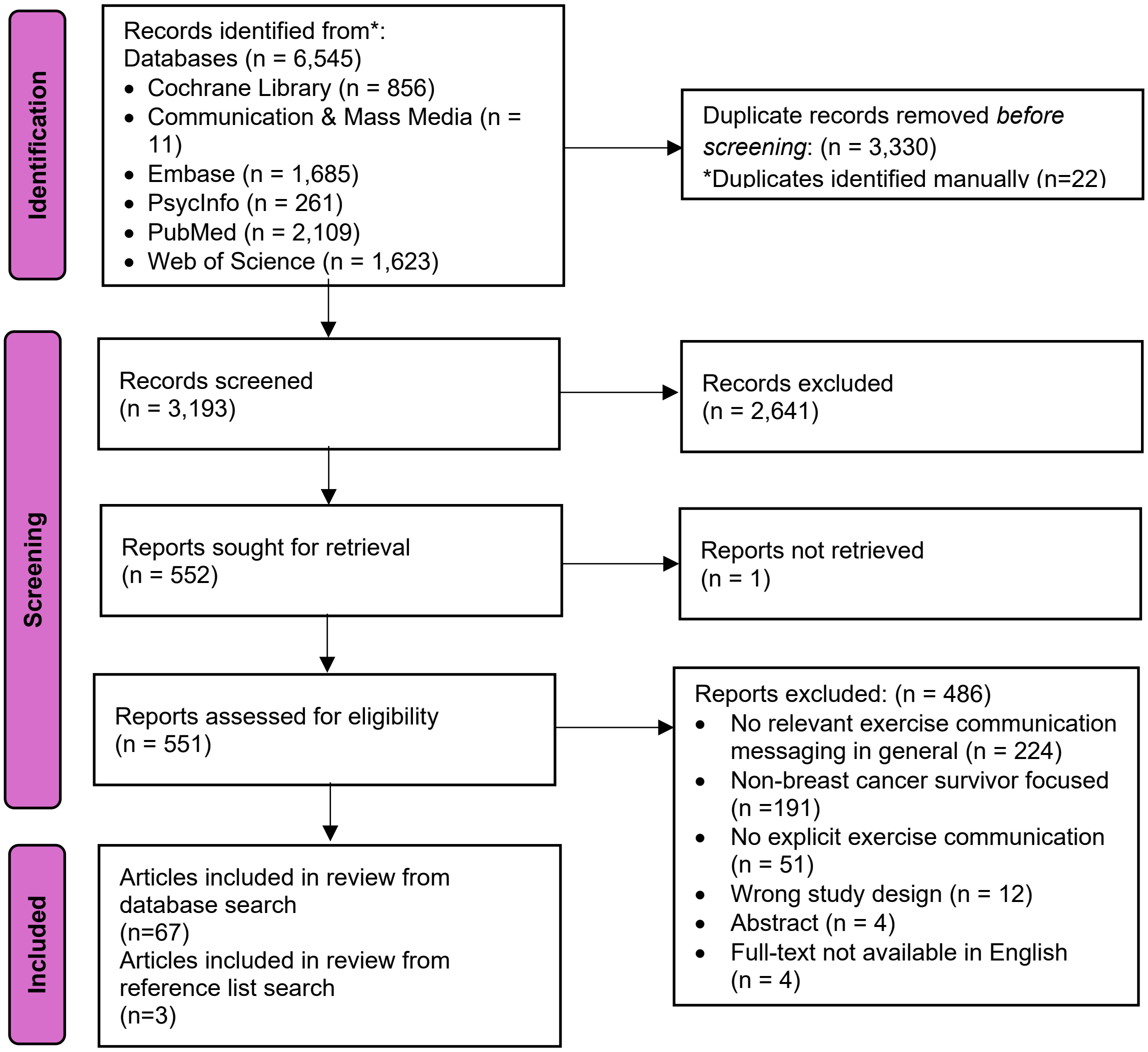

eFigure 1. Article Identification Process Using PRISMA Research Framework[1]

*No automation tool was used to remove additional records

© 2025 Wilson OWA et al. *JAMA Network Open*.

eAppendix 3. Study and Breast Cancer Survivor Characteristics

Most of the 39 studies were conducted in the U.S. (n=21, 53.8%). Other countries included Australia (n=3, 7.7%), Belgium (n=1, 2.6%), Canada (n=4, 10.3%), China (n=1, 2.6%), England (n=1, 2.6%), France (n=2, 5.1%), Spain (n=1, 2.6%), South Korea (n=3, 7.7%), and Sweden (n=1, 2.6%). The first article was published in 2007, 65 of the 70 articles were published from 2009 onwards, and 25 were published within the past five years. Most studies reported the results of a randomized controlled trial (RCT) or intervention (n=30, 76.9%); five (12.8%) reported on intervention development,[12-19] three (7.7%) were purely descriptive,[20-23] and one reviewed electronic health records (2.6%).[24]

Breast cancer survivor characteristics are summarized in eAppendix 3. The average age and body mass index of survivors were 55.5 years and 31.4 kg/m^2 , respectively. The studies predominantly included Non-Hispanic White and post-menopausal women (see eAppendix 3 demographic characteristic details). The average time since diagnosis was 2.9 years, most were diagnosed with stage I (Md: 32.2%) through II (Md: 37.5%) cancer, nearly all had received some form of surgery (Md: 98.8%), and most had received chemotherapy (Md: 66.7%), hormone therapy (Md: 63.2%), or radiotherapy (Md: 75.0%) (see eTable 4 for clinical characteristics details). Most survivors had an education equivalent to a college graduate or greater (Md: 50.7%), were employed (Md: 53.8%), and were married, in de facto relationship, or living with a partner (Md: 74.4%). Income was reported by eight studies, though results are not comparable across studies. Only five studies reported on urbanicity/rurality. Two reported that most survivors resided in rural areas,[25-28] another reported that 29.7% were rural residents,[29] and the vast majority did not reside in a rural area in the remaining two.[23,30-38] Details on participant contextual characteristics are reported in eTable 5.

© 2025 Wilson OWA et al. *JAMA Network Open*.

eTable 3. Participant Characteristics

Characteristic	Studies reporting (n)	Md (Range; SE)
**Demographic**		
**Age**	34	55.5 years (41.0-67.0 years; 1.00)
Race/ethnicity		
Asian	9	21.8% (0.6-100%; 0.14)
Black	14	16.7% (1.7-100%; 0.10)
Hispanic	9	7.0% (0.9-100%; 0.09)
Indigenous	2	0.6% (0.6-0.6%; 0.05)
Pasifika	1	0.3
White	18	85.5% (47.4-98.7%; 0.08)
Other*	15	5.0% (1.2-23.1%; 0.05)
Weight status	20	31.4kg/m^2 (23.9-36.1kg/m^2; 0.77)
Menopausal status		
Postmenopausal	14	80.1% (24.4-100%; 0.10)
**Clinical**		
Stage		
0	27	0.0% (0-25.8%; 0.00)
I	27	32.2% (0-62.8%; 0.09)
II	27	37.5% (0-59.0%; 0.10)
III	26	13.5% (0-43.3%; 0.07)
IV	26	0.0% (0-100%; 0.00)
Treatment		
Chemotherapy	23	66.7% (29.0-100%; 0.10)
Hormone therapy	19	63.2% (16.7-100%; 0.11)
Radiotherapy	19	75.0% (53.0-100%; 0.10)
Surgery (general)	12	98.8% (72.4-100%; 0.03)
Surgery (mastectomy)	7	48.0% (28.3-81.0%; 0.19)
Targeted therapy	1	15.4%
Immunotherapy	1	20.6%
Time since diagnosis	14	3.1 years (0.4-8.4 years; 0.66)
**Contextual**		
Education		
<High School	14	11.0% (0-41.0%; 0.08)
≥High school-<College graduate	16	28.1% (11.0-73.3%; 0.11)
≥College graduate	22	50.7% (25.5-77.0%; 0.11)
Employment		
Some form of employment	13	53.8% (20.5-76.0%; 0.14)
Marital status		
Married/partnered	18	74.4% (18.8-91.0%; 0.10)

Notes. *The definition of the ‘other’ race and ethnicity group differed across studies. Md = Median, SE = Standard Error

© 2025 Wilson OWA et al. *JAMA Network Open*.

eTable 4. Participant Demographic Characteristics

Country - Study name or setting	Age (years)	Race/ethnicity				Weight status (M (Range or SD)	Menopausal status
		Asian	Black	White	Other*		
Australia - NSW (EMPOWER)[39,40]	53.8 (9.6)	21.8%	-	47.4%	23.1%	33.7kg/m^2 (27.6-39.9)	-
Australia - QLD (Living Well after Breast Cancer)[41-43]	55.9 (9.1)	-	-	98.7%	1.3%	31.4kg/m^2 (4.9)	57% Post
Australia - National (Move More for Life Trial)[26-28]	55.5 (35-78)	-	-	-	-	27.2kg/m^2 (5.2)	85.5% Post
Australia, Germany, Netherlands, Spain, Sweden (EFFECT)[17]	≥18	N/A – Methodological paper, no data collected					
Belgium (Step*By*Step)[44]	41 (8)	-	-	-	-	-	-
Canada - AB (ACTION)[45-47]	58 (30-90)	-	-	-	-	27.7kg/m^2 (5.6)	62.0% Post
Canada - ON (LISA Trial)[48,49]	61.6 (6.7)	1.8%	2.3%	94.2%	0.6% Native American; 1.2% Other	31.4kg/m^2 (5.0)	100% Post
Canada - National (MatchQEP)[16]	≥18	N/A – Methodological paper, no data collected					
Canada - AB (PROACTIVE)[29]	52.8 (9.8)	-	-	95%	5%	66.2kg (10.0)	45% Post
China (WMFHAB)[50]	47.2 (6.0)	Chinese (predominantly)	-	-	-	50.0% ~24-28kg/m^2<br>50.0% ≥28kg/m^2	-
England (PHAB)[51,52]	52.4 (10.3)	-	3%	97%	-	68.7kg (10.5)	81% Post
France (Falzon et al. 2015)[53]	57.1 (12.1)	-			-	-	-
France (Martin et al. 2021)[23]	46.4 (8.4)	-			-	-	-
South Korea (Kong et al. 2021)[54]	47.1 (7.9)	South Korean (predominantly)	0%	0%	0%	23.9kg/m^2 (3.3)	-
South Korea (Smart After Care)[55,56]	50.4 (9.5)	South Korean (predominantly	0%	0%	0%	-	-
South Korea (WSEDI)[57]	41.5 (6.3)	South Korean (predominantly)	0%	0%	0%	66.7% >18.5-<23 kg/m^2	-
Spain (Travier and colleagues)[58,59]	55.1 (8.3)	-	-	-	-	30.5kg/m^2 (3.9)	62.2% Post
Sweden (Bergqist et al. 2021)[22]	41-78	Not measured	-	-	-	Not measured	-
U.S. - TX (ALIVE)[60]	52.2 (8.6)	0%	83%	0%	11% Hispanic<br>6% Mixed	30.8kg/m^2 (6.0)	-
U.S. - AL, IL (BEAT Cancer)[61,62]	54.9 (9.3)	0%	11.3%	83.8%	2.7% Hispanic<br>4.9% Other	-	84.6% Post
U.S. - NC, TX (DAMES)[63,64]	61.3 (7.4)	1%	18%	74%	7% Hispanic	31.0kg/m^2 (2.6)	100% Post
U.S. - AL, CA, CO, MO (ENERGY)[65-67]	56.1 (9.4)	1.5%	10.4%	83.2%	0.6% American Indian; 0.3% Hawaiian/Pacific Islander; 3.8% Mixed/Other	31.6kg/m^2 (4.7)	85.8% Post
U.S. - National (Fit2Thrive)[12-15]	52.5 (9.9)	-	5.9%	87.0%	6.7% Hispanic<br>7.1% Other	28.0kg/m^2 (6.3)	-
U.S. - CA (I Can!)[68]	≥40	N/A – Methodological paper, no data collected					
U.S. - CT (LEAN)[69,70]	59.0 (7.5)	-	-	91%	9%	33.1kg/m^2 (6.6)	82% Post
U.S. - CA (Memory and Motion)[71-73]	58.2 (11.4)	-	-	83.7%	18.6% Hispanic; 16.3% non-White	26.7kg/m^2 (6.2)	-
U.S. - NY (Mi Vida Saludable)[18,19]	56.8 (9.9)	-	-	-	100% Hispanic	≥18.5-<25.0kg/m^2 (=17.2%; ≥25.0-<30. 0kg/m^2=44.8%; ≥30.0kg/m^2 =38.0%	-
U.S. - TX (MOCHA)[74]	57 (9)	-	-	-		32.7kg/m^2 (5.7)	-
U.S. - GA, NC (Reach)[30-38]	57.3 (10.8)	0.6%	16.7%	81.4%	1.2% Other; 1.9% Hispanic	-	-
U.S. - NJ (Spark People)[75]	61.5 (8.8)	-	100%	-	-	36.7kg/m^2 (8.8)	-
U.S. - NC (STRENGTH trial)[76]	41.8 (5.6)	-	12%	85%	3%	25.8kg/m^2 (6.1)	100% Pre
U.S. - DC (The Stepping STONE Study)[77]	54.7 (9.8)	-	100%	-	-	35.2kg/m^2 (4.8)	-
U.S. - NC (WELL Body Study)[78]	52.4 (8.3)	-	100%	-	-	33.3kg/m^2 (5.7)	79.2% Post
U.S. - NC (WWE)[20,21]	67 (46-87)	-	-	86%	14%	29kg/m^2 (19-41)	-
U.S. - NC, TX (Nyrop et al. 2020)[24]	56.5 (25-86)	-	37%	58%	5%	36kg/m^2 (30-59)	-
U.S. - KS (Fazzino et al. 2017[25]	58.6 (8.0)	-	-	97%	3%	33.7kg/m^2 (4.0)	-
U.S. - MA (Ligibel et al. 2010)[79]	47.0 (7.3)	-	-	-	-	-	24.4% Post
U.S. - CT (Park et al. 2016)[80]	56.7 (10.8)	-	1.7%	95.7%	0.9% Hispanic; 1.7% Other	-	-
U.S. - MA(Quintiliani et al. 2016)[81]	58.6 (6.1)	-	30%	50%	10% Hispanic; 10% Other	-	-

Notes. With the exception of one study that used electronic health records,[24] race and ethnicity data were self-reported by survivors; *The definition of the 'other' race and ethnicity group differed across studies; M = Mean; SD = Standard Deviation; ALIVE = A Lifestyle Intervention Via Email; DAMES = Daughters and Mothers Against Breast Cancer; ENERGY = Exercise and Nutrition to Enhance Recovery and Good Health for You; I Can! = Improving Cognition After Cancer; MOCHA = Methodist Hospital Cancer Health Application; PHAB = A Home-based Physical Activity Intervention in Breast Cancer Survivors; PROACTIVE = Promoting Physical Activity during Chemotherapy; STRENGTH = Survivor Training for Enhancing Total Health; WSEDI = Web-based self-management exercise and diet intervention; WWE = Walking With Ease; WMFHAB = Weight Management for Health and Beauty; NA = Not applicable; kg/m^2 = kilograms per meter squared; Post = Postmenopausal; - = Not reported

eTable 5. Participant Clinical Characteristics

Country (Study name or authors)	Stage					Treatment (%)					Time since diagnosis in years (M (SD)
	0	I	II	III	IV	CT	HT	RT	Surg	Other	
Australia - NSW (EMPOWER)[39,40]	0%		100%		0%	62.3%	66.7%	88.5%	100%	15.4% TT	-
Australia - QLD (Living Well after Breast Cancer)[41-43]	0%	51%	38%	11%	0%	61%	59%	80%	38% (M)	-	0.9 (0.4)
Australia - National (Move More for Life Trial)[26-28]	2.7%	22.4%	35.2%	21.0%	1.4%	67.6%	55.7%	68.5%	91.8%	-	-
Australia, Germany, Netherlands, Spain, Sweden (EFFECT)[17]	0%	0%	0%	0%	100%	-	-	-	-	-	-
Belgium (Step*By*Step)[44]			-			-	-	-	-	-	-
Canada - AB (ACTION)[45-47]	0%	51.5%	42.7%	5.8%	0%	53.9%	66.8%	69.2%	100%	-	3.3 (0.9)
Canada - ON (LISA Trial)[48,49]	0%	22.2%	56.1%	21.6%	0%	56.1%	100%	81.3%	28.3% (M)	-	0.8
Canada - National (MatchQEP)[16]	N/A – Methodological paper, no data collected										
Canada - AB (PROACTIVE)[29]	0%	16%	58%	26%	0%	100% (ACT)	-	-	-	-	-
China (WMFHAB)[50]	3.3% 0	16.7%	40.0%	13.3%	0%	100%	73.3%	100%	100%	-	-
England (PHAB)[51,52]	0%		100%		0%	52.5%	-	-	-	-	0.8 (0.5)
France (Falzon et al. 2015)[53]	0%		100%		0%	-	-	-	-	-	-
France (Martin et al. 2021)[23]			-			100%	88.9%	88.9%	55.6% (M)	-	-
South Korea (Kong et al. 2021)[54]	0%	18.4%	40.8%	40.8%	0%	66.4%	63.2%	100%	72.4%	-	-
South Korea (Smart After Care)[55,56]	6.2%	35.1%	32.2%	13.6%		64.3%	-	68.4%	98.8%	-	-
South Korea (WSEDI)[57]	0%	6.7%	40.0%	43.3%	10.0%	86.7%	-	90.0%	33.3% (M)	-	<2
Spain (Travier and colleagues)[58,59]	0%	45.9%	43.2%	10.8%	0%	-	-	-	-	-	-
Sweden (Bergqist et al. 2021)[22]			-			-	100% (ACT)	-	-	-	-
U.S. - TX (ALIVE)[60]	20%		54%		26%	75%	44%	69%	94%	-	8.4 (6.5)
U.S. - AL, IL (BEAT Cancer)[61,62]	11.8%	42.7%	33.6%	11.8%	0%	53.6%	49.1%	71.8%	-	-	4.8 (4.5)
U.S. - NC, TX (DAMES)[63,64]	18%	43%	31%	4%	0%		Completed primary treatment				2.0 (1.1)
U.S. - AL, CA, CO, MO (ENERGY)[65-67]	0%	32.2%	48.4%	19.4%	0%	77%	54.6%	-	-	-	≤5
U.S. - National (Fit2Thrive)[12-15]	0%	39.0%	42.0%	16.0%	0%		≥3 months since primary treatment				-
U.S. - CA (I Can!)[68]	0%		100%		0%	-	-	-	-	-	-
U.S. - CT (LEAN)[69,70]	15%	51%	24%	7%	0%	29%	78%	53%	100%	-	2.9 (2.1)
U.S. - CA (Memory and Motion)[71-73]	0%	62.8%	27.9%	9.3%	0%	53.5%	72.1%	?	100%	-	<5
U.S. - NY (Mi Vida Saludable)[18,19]	24.6%	30.5%	18.6%	10.8%	0%	61.7%	47.3%	63.5%	98.8%	-	6.0 (4.9)
U.S. - TX (MOCHA)[74]	0%		100%		0%		Not in active treatment				-
U.S. - GA, NC (Reach)[30-38]	16.1%	46.6%	27.3%	9.9%	0%		-				2.6 (1.4)
U.S. - NJ (Spark People)[75]	41.9%		35.5%	22.6%	0%	77.1%	57.1%	68.6%	97.1%	20.6% IT	6.6 (4.3)
U.S. - NC (STRENGTH trial)[76]	0%	33%	59%	8%	0%	100% (ACT)	50%	-	-	-	-
U.S. - DC (The Stepping STONE Study)[77]	25.8%	32.3%		-			0.5-5 years post-active treatment				-
U.S. - NC (WELL Body Study)[78]	0%		25.0		50.0%	25.0%	0%	79.2%	16.7%	83.3%	95.8%
U.S. - NC (WWE)[20,21]	0%		100%		0%	67%	100% (ACT)	75%	81% (M)	-	-
U.S. - NC, TX (Nyrop et al. 2020)[24]	0%	27%	46%	25%	0%	65%	80%	76%	48% (M)	-	-
U.S. - KS (Fazzino et al. 2017[25]	9%	41%	37%	12%	0%		-				~5
U.S. - MA (Ligibel et al. 2010)[79]	0%	26.8%	36.6%	34.2%	0%	97.6%	-	53.7%	56.1% (M)	-	0.4 (0.2)
U.S. - CT (Park et al. 2016)[80]	0%	100%		0%	0%		Completed primary treatment in <3 months				<1.5
U.S. - MA(Quintiliani et al. 2016)[81]			-				6 months post treatment				-

Notes. M = Mean; SD = Standard Deviation; ALIVE = A Lifestyle Intervention Via Email; DAMES = Daughters and Mothers Against Breast Cancer; ENERGY = Exercise and Nutrition to Enhance Recovery and Good Health for You; I Can! = Improving Cognition After Cancer; MOCHA = Methodist Hospital Cancer Health Application; PHAB = A Home-based Physical Activity Intervention in Breast Cancer Survivors; PROACTIVE = Promoting Physical Activity during Chemotherapy; STRENGTH = Survivor Training for Enhancing Total Health; WSEDI = Web-based self-management exercise and diet intervention; WWE = Walking With Ease; WMFHAB = Weight Management for Health and Beauty; WELL Body = Weighing Every day for Love of Life and Body; ACT = Active treatment; CT = Chemotherapy; HT = Hormone therapy; RT = Radiotherapy; Surg = Surgery; TT = Targeted therapy; IT = Immunotherapy; M = Mastectomy; - = Not reported.

© 2025 Wilson OWA et al. *JAMA Network Open*.

eTable 6. Participant Contextual Characteristics

Country - Study name or setting	Education			Annual income	Employment	Marital status
	<HS	≥HS-<College	≥College			
Australia - NSW (EMPOWER)[39,40]	34.3%	28.9%	36.8%	-	65.3% FT/PT	64.9% Married/DeFacto
Australia - QLD (Living Well after Breast Cancer)[41-43]	41%	20%	39%	41.8% ≥82K AUD	63.3% FT/PT/Casual	68.4% Married/DeFacto
Australia - National (Move More for Life Trial)[26-28]	-	-	46.6%	38.8% >52K AUD	20.5% FT	77.2% Married/DeFacto
Australia, Germany, Netherlands, Spain, Sweden (EFFECT)[17]	-	-	-	-	-	-
Belgium (Step*By*Step)[44]	-	-	-	-	-	-
Canada - AB (ACTION)[45-47]	-	-	29.7%	26.3% >80K CAD	30.2% FT	72.1% Married
Canada - ON (LISA Trial)[48,49]	-	-	-	-	-	71.9% Married
Canada - National (MatchQEP)[16]	N/A – Methodological paper, no data collected					
Canada - AB (PROACTIVE)[29]	22%		77%	-	43% FT/PT	83.7% Married
China (WMFHAB)[50]	0%	73.3%	26.7%	-	-	-
England (PHAB)[51,52]	-	-	38%	-	53% FT/PT	91% Married/DF
France (Falzon et al. 2015)[53]	1.9%	40.4%	57.7%	-	-	69.2% Married/DF
France (Martin et al. 2021)[23]	-	-	-	-	-	66.7% Married
South Korea (Kong et al. 2021)[54]	-	-	54.6%	-	-	80.3% Married
South Korea (Smart After Care)[55,56]	-	-	?	-	-	.
South Korea (WSEDI)[57]	-	-	76.7%	-	-	90% married
Spain (Travier and colleagues)[58,59]	-	-	-	-	-	81.0% Married/relationship
Sweden (Bergqist et al. 2021)[22]	Not measured			-	-	-
U.S. - TX (ALIVE)[60]	-	-	65%	-	76%	-
U.S. - AL, IL (BEAT Cancer)[61,62]	15.7 years (2.7)			71.6% ≥50K USD	69.1%	68.2% Married/cohabitating
U.S. - NC, TX (DAMES)[63,64]	1.5%	64.2%	34.3%	69% ≥40K USD	-	-
U.S. - AL, CA, CO, MO (ENERGY)[65-67]	15.6 years (2.5)			-	-	-
U.S. - National (Fit2Thrive)[12-15]	-	-	-	-	-	-
U.S. - CA (I Can!)[68]	-	-	-	-	-	-
U.S. - CT (LEAN)[69,70]	0%	34%	66%	-	-	74.4% Married/cohabitating
U.S. - CA (Memory and Motion)[71-73]	32.7%		67.5%	-	-	-
U.S. - NY (Mi Vida Saludable)[18,19]	27.5%	47.0%	25.5%	11.9% ≥60K USD	22.2% FT	-
U.S. - TX (MOCHA)[74]	-	-	-	-	-	-
U.S. - GA, NC (Reach)[30-38]	5.0%	27.3%	67.7%	-	69.5% FT/PT	-
U.S. - NJ (Spark People)[75]	48.6%		51.4%	-	35.3%	18.8% Married
U.S. - NC (STRENGTH trial)[76]	-	-	-	-	-	-
U.S. - DC (The Stepping STONE Study)[77]	-	51.6%		-	-	-
U.S. - NC (WELL Body Study)[78]	25.0%		75.0%	50.0% ≥60K USD	53.8% FT	53.8% Married
U.S. - NC (WWE)[20,21]	30% ≤HS		-	-	-	-
U.S. - NC, TX (Nyrop et al. 2020)[24]	Not measured			-	-	-
U.S. - KS (Fazzino et al. 2017[25]	0%	61%	39%	-	-	76% Married/cohabitating
U.S. - MA (Ligibel et al. 2010)[79]	-	-	?	-	-	-
U.S. - CT (Park et al. 2016)[80]	0.9%	38.3%	60.9%	72.7% ≥60K USD	-	78.8% Married/relationship
U.S. - MA(Quintiliani et al. 2016)[81]	50%		50%	-	66.7%	-

Notes. ALIVE = A Lifestyle Intervention Via Email; DAMES = Daughters and Mothers Against Breast Cancer; ENERGY = Exercise and Nutrition to Enhance Recovery and Good Health for You; I Can! = Improving Cognition After Cancer; MOCHA = Methodist Hospital Cancer Health Application; PHAB = A Home-based Physical Activity Intervention in Breast Cancer Survivors; PROACTIVE = Promoting Physical Activity during Chemotherapy; STRENGTH = Survivor Training for Enhancing Total Health; WSEDI = Web-based self-management exercise and diet intervention; WWE = Walking With Ease; WMFHAB = Weight Management for Health and Beauty; WELL Body = Weighing Every day for Love of Life and Body; HS = High school; NA = Not applicable; AUD = Australian dollars; CAD = Canadian dollars; USD = United States dollars; FT = Full-time; PT = Part-ti

© 2025 Wilson OWA et al. *JAMA Network Open*.

eTable 7. Intervention Characteristics and Nonexercise Communication Components

Country - Study name or setting	Intervention / follow-up length	Theoretical framework	Non-exercise communication component(s)	Additional exercise intervention component(s)
Australia - NSW (EMPOWER)[39,40]	6 month intervention	SCT	Diet; medication adherence and side effects; social or emotional wellbeing; and, general breast cancer information	-
Australia - QLD (Living Well after Breast Cancer)[41-43]	12 month intervention; 6 month follow-up	SCT	Diet	Pedometer, scale, measuring tape, and monitoring materials
Australia - National (Move More for Life Trial)[26-28]	12 week intervention	SCT	-	-
Australia, Germany, Netherlands, Spain, Sweden (EFFECT)[17]	9 month intervention	-	-	Multi-modal supervised exercise program that covered balance, aerobic, and muscle-strengthening exercise. Encouragement to participate in unsupervised exercise
Belgium (Step*By*Step)[44]	NA – Intervention development	TPB, TTM	-	Pedometer
Canada - AB (ACTION)[45-47]	One off intervention; 4 and 12 week follow-ups	TPB	-	Pedometer
Canada - ON (LISA Trial)[48,49]	24 month intervention	SCT	Diet; breast cancer; therapy compliance; osteoporosis; and, other general medical issues	Goals; Two year subscription to Canadian Health Magazine
Canada - National (MatchQEP)[16]	10 week intervention; 12 week follow-up	-	-	Fitbit. MatchQEP survivors received tailored exercise programs, Match survivors received generic exercise programs
Canada - AB (PROACTIVE)[29]	6 month intervention	TPB	-	Pedometer and step logbook
China (WMFHAB)[50]	12 week intervention	Self-determination theory	Diet; weight management guidance	-
England (PHAB)[51,52]	6 month intervention	-	Basic lifestyle information	-
France (Falzon et al. 2015)[53]	One off	TPB	-	-
France (Martin et al. 2021)[23]	NA - Descriptive study	.	-	Pedometer
South Korea (Kong et al. 2021)[54]	5 week intervention	TTM	-	Fitbit and Smartphone app
South Korea (Smart After Care)[55,56]	12 week intervention	-	-	Pedometer and Smartphone app
South Korea (WSEDI)[57]	12 week intervention	TTM	Diet	-
Spain (Travier and colleagues)[58,59]	12 week intervention	-	Diet; weight	Pedometer
Sweden (Bergqist et al. 2021)[22]	NA - Descriptive study	-	Drug compliance; self-care activities; smoking; treatment; and, symptoms	-
U.S. - TX (ALIVE)[60]	3 month intervention	SCT, TTM, goal-setting theory, social marketing,	-	-
U.S. - AL, IL (BEAT Cancer)[61,62]	3 month intervention; 3 month follow-up	SCT	Diet	-
U.S. - NC, TX (DAMES)[63,64]	12 month intervention	SCT	Diet	-
U.S. - AL, CA, CO, MO (ENERGY)[65-67]	4 year intervention	SCT	Diet; weight	Pedometer
U.S. - National (Fit2Thrive)[12-15]	12 week intervention; 12 week follow-up	SCT	-	Fitbit and Smartphone app
U.S. - CA (I Can!)[68]	6 month intervention; 6 month follow-up	SCT	-	Fitbit
U.S. - CT (LEAN)[69,70]	6 month intervention	SCT	-	Fitbit and journal
U.S. - CA (Memory and Motion)[71-73]	3 month intervention	SCT, TTM	-	Fitbit
U.S. - NY (Mi Vida Saludable)[18,19]	12 month intervention; 6 month follow-up	SCT	Diet	-
U.S. - TX (MOCHA)[74]	4 week intervention	-	Diet; Wellness	Fitbit
U.S. - GA, NC (Reach)[30-38]	9 month intervention; 3 month follow-up	SCT, TTM	-	-

© 2025 Wilson OWA et al. *JAMA Network Open*.

Country - Study name or setting	Intervention / follow-up length	Theoretical framework	Non-exercise communication component(s)	Additional exercise intervention component(s)
U.S. - NJ (Spark People)[75]	12 month intervention; 6 month follow-up	SCT	Diet; weight	Fitbit
U.S. - NC (STRENGTH trial)[76]	6 month intervention	SCT	Diet	Videotape, exercise equipment
U.S. - DC (The Stepping STONE Study)[77]	12 week intervention	TPB	Diet	Pedometer and notebooks to track their behaviors
U.S. - NC (WELL Body Study)[78]	6 month intervention	Self-regulation theory	Diet; weight	Exercise tracker and scale
U.S. - NC (WWE)[20,21]	NA - Descriptive study	SCT	Aromatase inhibitor side effects	-
U.S. - NC, TX (Nyrop et al. 2020)[24]	NA - EHR review	-	Nutrition, weight	-
U.S. - KS (Fazzino et al. 2017[25]	6 month intervention; 12 month follow-up	-	Diet	Pedometer and self-monitoring charts
U.S. - MA (Ligibel et al. 2010)[79]	12 week intervention	-	-	Pedometer, exercise protocol, exercise log, and heart rate monitor
U.S. - CT (Park et al. 2016)[80]	4 month intervention; 3 month follow-up	-	Diet	-
U.S. - MA(Quintiliani et al. 2016)[81]	10 week intervention	-	Diet; sleep; and, fatigue	Pedometer and scale

Notes. ALIVE = A Lifestyle Intervention Via Email; DAMES = Daughters and Mothers Against Breast Cancer; ENERGY = Exercise and Nutrition to Enhance Recovery and Good Health for You; I Can! = Improving Cognition After Cancer; MOCHA = Methodist Hospital Cancer Health Application; PHAB = A Home-based Physical Activity Intervention in Breast Cancer Survivors; PROACTIVE = Promoting Physical Activity during Chemotherapy; STRENGTH = Survivor Training for Enhancing Total Health; WSEDI = Web-based self-management exercise and diet intervention; WWE = Walking With Ease; WMFHAB = Weight Management for Health and Beauty; WELL Body = Weighing Every day for Love of Life and Body; ACS = American Cancer Society; ACSM = American College of Sports Medicine; AICR = American Institute for Cancer Research; WCRF = World Cancer Research Fund; UK= United Kingdom; SCT = Social cognitive theory; TTM = Transtheoretical model; TPB = Theory of planned behavior

© 2025 Wilson OWA et al. *JAMA Network Open*.

eTable 8. Content of Exercise Information Communicated to Breast Cancer Survivors

Country (Study name or authors)	Aerobic exercise	Muscle-strengthening exercise	Source	Benefits/safety	Individualization
Australia - NSW (EMPOWER)[39,40]	10,000 steps/day	?	WHO (not specified)	Mental wellbeing	?
Australia - QLD (Living Well after Breast Cancer)[41-43]	To ≥210min/wk, 10,000 steps/day; ≥30min/day MPA	2-3session/wk	ACSM 2009[82]; WCRF/AICR 2018[83]	?	?
Australia - National (Move More for Life Trial)[26-28]	30min, on most days/wk	1-3 days/wk	Australian guidelines[84]	Recurrence, mortality, fitness, mental wellbeing[85]	Yes
Australia, Germany, Netherlands, Spain, Sweden (EFFECT)[17]	30min, 5days/wk; 150min/wk	?	Roundtable* 2019[86]	?	Yes
Belgium (Step*By*Step)[44]	10,000 steps/day	?	?	?	Yes
Canada - AB (ACTION)[45-47]	30min, 5 days/wk, 10,000 steps	?	?	Recurrence, mortality, fitness, mental wellbeing[85]	?
Canada - ON (LISA Trial)[48,49]	150-200min/wk MPA	Mentioned, not specified	?	?	Yes
Canada - National (MatchQEP)[16]	≥150min/wk MVPA	?	ACSM 2010[87]; Roundtable* 2019[86]; Cancer Care Ontario 2017[88]	Benefits communicated (no details reported); Stress, sleep covered	?
Canada - AB (PROACTIVE)[29]	≥150min/wk	?	?	Recurrence, mortality, fitness, mental wellbeing[85]	?
China (WMFHAB)[50]	Progression up to 4–5 day/wk MPA, 40–60 min/day	Progression up to 3 days/wk, 2 sets of 12–15 reps, 2-min rest between sets	Roundtable* 2019[86]; ACSM 2009[82]	Safety - why survivors can exercise	Yes
England (PHAB)[51,52]	30min MPA, 5-7days/wk	?	UK National Guidelines[89]	Benefits communicated (no details reported)	?
France (Falzon et al. 2015)[53]	Regular moderate intensity exercise	Mentioned, not specified	?	Fatigue, social ties, recurrence, rebuild muscle, self-image, help with daily tasks<br>Safety - No risk of lymphedema if supervised	?
France (Martin et al. 2021)[23]	6000 steps/day	?	?	Pain, tiredness, fatigue, fun	?
South Korea (Kong et al. 2021)[54]	150min/wk, 10,000 steps/day (up to 300min/wk, or 12,000 steps/day)	?	ACSM 2011[90]	?	Yes
South Korea (Smart After Care)[55,56]	150min/wk OR 90min/wk depending on physical function	Twice/wk, 4-8 major muscle groups, 2 sets of 10 repetitions	?	?	Yes
South Korea (WSEDI)[57]	150min/wk, self-adjust intensity duration and frequency	?	ACS 2006[91]	?	?
Spain (Travier and colleagues)[58,59]	10,000 steps	?	?	?	?
Sweden (Bergqist et al. 2021)[22]	150min/wk	?	WHO (unspecified)	Benefits communicated (no details reported)	?
U.S. - TX (ALIVE)[60]	150min/wk (MVPA)	?	Federal recommendations (unspecified)	?	?
U.S. - AL, IL (BEAT Cancer)[61,62]	150min/wk	?	ACS (unspecified)	Benefits communicated (no details reported)	?
U.S. - NC, TX (DAMES)[63,64]	150min/wk	Twice/wk strength training	ACS 2006[91]	?	Yes
U.S. - AL, CA, CO, MO (ENERGY)[65-67]	INTERVENTION: Start with 10min/day, incremental increases, building towards 60min MPA/day / 10,000 steps/day<br>CONTROL: 30min/day	2-3 sessions/wk	Institute of Medicine (unspecified)	?	Yes
U.S. - National (Fit2Thrive)[12-15]	Gradual increase to 150min/wk, plus step goals	?	?	Physical and psychological well-being, and chronic disease risk	Yes

© 2025 Wilson OWA et al. *JAMA Network Open*.

Country (Study name or authors)	Aerobic exercise	Muscle-strengthening exercise	Source	Benefits/safety	Individualization
U.S. - CA (I Can!)[68]	Goal to meet 150min/week of MVPA discussed	?	ACSM 2010[87]	?	Yes
U.S. - CT (LEAN)[69,70]	150min/wk MPA, 10,000 steps/day	?	ACS 2012[92]	Weight loss	?
U.S. - CA (Memory and Motion)[71-73]	150min/wk	?	ACSM 2010[87]	?	Yes
U.S. - NY (Mi Vida Saludable)[18,19]	30min, 5 days/wk; 150min/wk	?	ACS 2012[92]; AIRC/WCRF 2020[93]	Happiness	?
U.S. - TX (MOCHA)[74]	Personal messages regarding activity goals (see app images)	?	?	?	?
U.S. - GA, NC (Reach)[30-38]	150min/wk	?	National guidelines	?	Yes
U.S. - NJ (Spark People)[75]	30min/day; 10,000 steps/day	?	ACC/AHA/TOS 2014[94]	?	?
U.S. - NC (STRENGTH trial)[76]	≥30min/day, ≥ 3times/week	Every other day	Abernathy et al. 1994[95]	?	?
U.S. - DC (The Stepping STONE Study)[77]	Gradual progression to 10,000 steps/day	?	ACS 2006[91]	Risk-related information (details unclear)	Yes
U.S. - NC (WELL Body Study)[78]	Gradual increase to 150-225min/wk (30-45min/day on 5days/wk)	?	Ballard-Barbash et al. 2009[96]	Benefits outlined by cancer exercise guidelines (reduce your risk of recurrence, function better physically and emotionally, Improve quality of life, Reduce anxiety and depression Improve mood and boost self-esteem); Safety - information about staying safe when exercise unsupervised	Yes
U.S. - NC (WWE)[20,21]	150min/wk MPA, 30min, 5 days/wk	?	?	Joint pain reduction	?
U.S. - NC, TX (Nyrop et al. 2020)[24]	Progress towards ≥30min/day, 5 days/wk	?	?	Weight management	Yes
U.S. - KS (Fazzino et al. 2017[25]	225 min/wk of MVPA (gradually increase towards)	?	ACSM 2009[82]	?	?
U.S. - MA (Ligibel et al. 2010)[79]	150min/wk, 10,000 steps/day	?	?	?	?
U.S. - CT (Park et al. 2016)[80]	150min/wk	?	?	Benefits communicated (no details reported)	?
U.S. - MA(Quintiliani et al. 2016)[81]	10,000 steps/day	?	?	?	Yes

Notes. ALIVE = A Lifestyle Intervention Via Email; DAMES = Daughters and Mothers Against Breast Cancer; ENERGY = Exercise and Nutrition to Enhance Recovery and Good Health for You; I Can! = Improving Cognition After Cancer; MOCHA = Methodist Hospital Cancer Health Application; PHAB = A Home-based Physical Activity Intervention in Breast Cancer Survivors; PROACTIVE = Promoting Physical Activity during Chemotherapy; STRENGTH = Survivor Training for Enhancing Total Health; WSEDI = Web-based self-management exercise and diet intervention; WWE = Walking With Ease; WMFHAB = Weight Management for Health and Beauty; WELL Body = Weighing Every day for Love of Life and Body; ACS = American Cancer Society; ACSM = American College of Sports Medicine; AICR = American Institute for Cancer Research; WCRF = World Cancer Research Fund; UK= United Kingdom; MPA = moderate-intensity physical activity; moderate-to-vigorous intensity physical activity; min = minutes, wk = week; *Roundtable guidelines were officially endorsed by ACSM, ACS, AAPMR, ACLM, CSEP, CDC, CARF, ESSA, DVGS, MacMillan, NCCH, KVPD, SBM, Sunflower Wellness; ? = Unclear/not reported

© 2025 Wilson OWA et al. *JAMA Network Open*.

eTable 9. Why Exercise Information Was Communicated

Country (Study name or authors)	Why
Australia - NSW (EMPOWER)[39,40]	Increase exercise, manage treatment/medication side effects (e.g. fatigue, muscle soreness, etc.), improve mental wellbeing, connect with friends
Australia - QLD (Living Well after Breast Cancer)[41-43]	Weight loss
Australia - National (Move More for Life Trial)[26-28]	Increase exercise
Australia, Germany, Netherlands, Spain, Sweden (EFFECT)[17]	-
Belgium (Step*By*Step)[44]	-
Canada - AB (ACTION)[45-47]	Quality of life
Canada - ON (LISA Trial)[48,49]	Weight loss
Canada - National (MatchQEP)[16]	Increase exercise
Canada - AB (PROACTIVE)[29]	Increase exercise
China (WMFHAB)[50]	Weight management
England (PHAB)[51,52]	Increase exercise
France (Falzon et al. 2015)[53]	-
France (Martin et al. 2021)[23]	-
South Korea (Kong et al. 2021)[54]	-
South Korea (Smart After Care)[55,56]	-
South Korea (WSEDI)[57]	Health-related quality of life
Spain (Travier and colleagues)[58,59]	-
Sweden (Bergqist et al. 2021)[22]	Quality of life
U.S. - TX (ALIVE)[60]	-
U.S. - AL, IL (BEAT Cancer)[61,62]	-
U.S. - NC, TX (DAMES)[63,64]	Weight
U.S. - AL, CA, CO, MO (ENERGY)[65-67]	Weight loss
U.S. - National (Fit2Thrive)[12-15]	Increase exercise
U.S. - CA (I Can!)[68]	Cognitive functioning
U.S. - CT (LEAN)[69,70]	Weight loss
U.S. - CA (Memory and Motion)[71-73]	Cognitive functioning
U.S. - NY (Mi Vida Saludable)[18,19]	Lifestyle change
U.S. - TX (MOCHA)[74]	-
U.S. - GA, NC (Reach)[30-38]	Psychosocial outcomes (Quality of life, fatigue, physical functioning, mood)
U.S. - NJ (Spark People)[75]	Weight loss
U.S. - NC (STRENGTH trial)[76]	Body composition
U.S. - DC (The Stepping STONE Study)[77]	Increase exercise
U.S. - NC (WELL Body Study)[78]	Prevent weight gain
U.S. - NC (WWE)[20,21]	Reduce joint pain
U.S. - NC, TX (Nyrop et al. 2020)[24]	Weight management
U.S. - KS (Fazzino et al. 2017[25]	Weight loss or maintenance
U.S. - MA (Ligibel et al. 2010)[79]	Weight loss
U.S. - CT (Park et al. 2016)[80]	-
U.S. - MA (Quintiliani et al. 2016)[81]	-

Notes. ALIVE = A Lifestyle Intervention Via Email; DAMES = Daughters and Mothers Against Breast Cancer; ENERGY = Exercise and Nutrition to Enhance Recovery and Good Health for You; I Can! = Improving Cognition After Cancer; MOCHA = Methodist Hospital Cancer Health Application; PHAB = A Home-based Physical Activity Intervention in Breast Cancer Survivors; PROACTIVE = Promoting Physical Activity during Chemotherapy; STRENGTH = Survivor Training for Enhancing Total Health; WSEDI = Web-based self-management exercise and

diet intervention; WWE = Walking With Ease; WMFHAB = Weight Management for Health and Beauty; WELL Body = Weighing Every day for Love of Life and Body; - = Not reported

© 2025 Wilson OWA et al. *JAMA Network Open*.

eAppendix 4. Other Exercise Information Content

Considerations

In the MatchQEP study,[16] personal circumstances, including cancer-related characteristics, side effects, current fitness level, and available space and equipment were considered. Patient/survivor enjoyment were considered in the Weight Management for Health and Beauty study.[50] The ENERGY study[65-67] considered: convenience, enjoyment, time management, the environment, social support, overconcern with weight, problems and body image, showering, hair styling, and bathroom accessibility were also considered. The Stepping STONE study[77] accounted for concerns/motivations around physical activity, personal barriers, core values, and goals. Finally, Quintiliani et al.[81]considered influences on their behavior, such as finances, stress, family/friends/neighborhood.

Other content characteristics

Details were lacking regarding other characteristics of exercise communication content such as framing (gain vs. loss) and tone. One study compared tailored information (i.e. individualized communication informed by age, current exercise, health status, and outcome expectations) to targeted information (communication of information specifically on exercise for breast cancer survivors).[26-28] Another compared tailored information (plus a pedometer) to generic communication.[29]

© 2025 Wilson OWA et al. *JAMA Network Open*.

eTable 10. Format and Delivery of Exercise Information Communicated to Breast Cancer Survivors

Country - Study name or setting	Written communicated		Verbal communication	
	Form(s)	Who communicated	Form(s)	Who communicated
Australia - NSW (EMPOWER)[39,40]	Text	Researcher(s)	-	-
Australia - QLD (Living Well after Breast Cancer)[41-43]	Text	?	Phone	Dietician
Australia - National (Move More for Life Trial)[26-28]	Newsletter of booklet	?	-	-
Australia, Germany, Netherlands, Spain, Sweden (EFFECT)[17]	?	?	?	?
Belgium (Step*By*Step)[44]	Website	Medical staff	-	-
Canada - AB (ACTION)[45-47]	Guidebook	?	-	-
Canada - ON (LISA Trial)[48,49]	Workbook	?	Phone	Coach (lifestyle)
Canada - National (MatchQEP)[16]	Infographic	?	Video conference	Peer partner or qualified exercise professional
Canada - AB (PROACTIVE)[29]	Guidebook	?	-	.
China (WMFHAB)[50]	WeChat	?	Face-to-face	Researcher(s)
England (PHAB)[51,52]	Leaflets	?	Face-to-face	Researcher(s)
France (Falzon et al. 2015)[53]	Testimony	?	Face-to-face	Researcher(s)
France (Martin et al. 2021)[23]	?	?	?	?
South Korea (Kong et al. 2021)[54]	App	?	Face-to-face, Phone	Exercise physiologist
South Korea (Smart After Care)[55,56]	?	?	?	?
South Korea (WSEDI)[57]	?	?	?	?
Spain (Travier and colleagues)[58,59]	-	-	?	?
Sweden (Bergqist et al. 2021)[22]	App	?	-	-
U.S. - TX (ALIVE)[60]	?	?	?	?
U.S. - AL, IL (BEAT Cancer)[61,62]	Education notebook	?	Face-to-face	?
U.S. - NC, TX (DAMES)[63,64]	Workbook	?	-	-
U.S. - AL, CA, CO, MO (ENERGY)[65-67]	Email, print materials	?	Phone	?
U.S. - National (Fit2Thrive)[12-15]	App, packet, text	?	Phone	Coach
U.S. - CA (I Can!)[68]	-		Face-to-face, Phone	Coach (health)
U.S. - CT (LEAN)[69,70]	Book, Brochure	?	Face-to-face, Phone	Dietician
U.S. - CA (Memory and Motion)[71-73]	-	-	Phone	Clinical psychologist
U.S. - NY (Mi Vida Saludable)[18,19]	Email, Newsletter, Text	?	Face-to-face	Researchers(s) (Study staff)
U.S. - TX (MOCHA)[74]	App	?	-	-
U.S. - GA, NC (Reach)[30-38]	Text	?	Phone	?
U.S. - NJ (Spark People)[75]	Handout	?	-	-
U.S. - NC (STRENGTH trial)[76]	Workbook	?	Phone, videotape	Researchers(s) (Study staff)
U.S. - DC (The Stepping STONE Study)[77]	-	-	Phone	Coach (trained survivor)
U.S. - NC (WELL Body Study)[78]	Email	Researcher(s)	Face-to-face	Researcher(s)
U.S. - NC (WWE)[20,21]	Brochure	Researcher(s)	-	-
U.S. - NC, TX (Nyrop et al. 2020)[24]	Handout	?	Face-to-face	Physician, Nurse Practitioner, or Physician Assistant
U.S. - KS (Fazzino et al. 2017[25]	-	-	Phone	?
U.S. - MA (Ligibel et al. 2010)[79]	-	-	Face-to-face, Phone	Exercise physiologist
U.S. - CT (Park et al. 2016)[80]	Materials	?	-	-
U.S. - MA(Quintiliani et al. 2016)[81]	?	?	?	?

Notes. ALIVE = A Lifestyle Intervention Via Email; DAMES = Daughters and Mothers Against Breast Cancer; ENERGY = Exercise and Nutrition to Enhance Recovery and Good Health for You; I Can! = Improving Cognition After Cancer; MOCHA = Methodist Hospital Cancer Health Application; PHAB = A Home-based Physical Activity Intervention in Breast Cancer Survivors; PROACTIVE = Promoting Physical Activity during Chemotherapy; STRENGTH = Survivor Training for Enhancing Total Health; WSEDI = Web-based self-management exercise and diet intervention; WWE = Walking With Ease; WMFHAB = Weight Management for Health and Beauty; WELL Body = Weighing Every day for Love of Life and Body; ? = Unclear; - = Not reported

eTable 11. Intervention Outcomes and Effects

Country - Study name or setting	Intervention outcomes and effects
Australia - NSW (EMPOWER)[39,40]	Exercise increased, BMI decreased, QOL improved, self-efficacy improved. No change in mental health symptoms, or other anthropometric variables. No difference from control group who received usual medical care
Australia - QLD (Living Well after Breast Cancer)[41-43]	Anthropometric variables, metabolic syndrome risk factors, QOL (physical and mental), musculoskeletal pain, and body image, and psychological menopause symptoms all improved. There was no difference in adverse events between intervention and the control group who received usual care, and not all improvements were due to the intervention
Australia - National (Move More for Life Trial)[26-28]	Tailored and targeted messaging were both associated with a small increase in aerobic and muscle-strengthening exercise, meeting aerobic guidelines compared to standard care. Both also had a moderate to large association with meeting muscle-strengthening guidelines. Tailored messaging had a small association with the FACT-B (QOL) and FACIT-F (fatigue) scales, whereas targeted messaging had not association with FACT-B (QOL), but a strong association with FACIT-F (fatigue)
Australia, Germany, Netherlands, Spain, Sweden (EFFECT)[17]	NA - Methods only
Belgium (Step*By*Step)[44]	NA - Communication usability was the focus
Canada - AB (ACTION)[45-47]	Exercise increased; QOL did not change. Intention, attitude, and planning were greater in the intervention group. Intention and planning partially mediated the effects of the intervention on exercise, Increases in exercise were not maintained post-intervention
Canada - ON (LISA Trial)[48,49]	Those who received goals (intervention) in addition to information only (comparison) decreased their weight without adverse effects on QOL, hospitalizations, or medical events, and also had greater rates of disease free survival at 8 years follow up. Physical QOL improved, whereas no change was found for mental QOL
Canada - National (MatchQEP)[16]	NA - Methods only
Canada - AB (PROACTIVE)[29]	Tailored print materials plus a pedometer and step log book were no more effective than standard public health guidelines at increasing exercise
China (WMFHAB)[50]	Exercise increased; anthropometric variables improved; grip strength increased<br>An intervention effect was only observed for changes in exercise and grip strength in comparison to a group who received information about weight management and exercise compared to the fortnightly exercise prescriptions (intervention group)
England (PHAB)[51,52]	Exercise increased; HRQOL, functional wellbeing, and VO2 max improved; body mass, BMI, and LDL-C decreased<br>Only changes in exercise and VO2 max were different from the control group who received standard care
France (Falzon et al. 2015)[53]	Exercise self-efficacy and intention were higher in those who received narratives/testimony than the expert recommendations group, whereas beliefs about benefits and risks were more favorable among the expert recommendation group
France (Martin et al. 2021)[23]	NA - Qualitative evaluation of intervention
South Korea (Kong et al. 2021)[54]	The addition of a Fitbit and Smartphone app increased exercise more compared to exercise information alone
South Korea (Smart After Care)[55,56]	The addition of a pedometer and Smartphone app increased exercise more compared to exercise information alone
South Korea (WSEDI)[57]	Exercise increased, and physical functioning fatigue, and motivational readiness all improved. Self-efficacy for exercise management improvement was greater in the intervention group<br>Improvements were greater compared to the control group
Spain (Travier and colleagues)[58,59]	Anthropometric variables, cardiorespiratory fitness, physical functioning, general health, mental health, and global health status, metabolic risk biomarkers, and insulin resistance indicators all improved<br>There was no control group
Sweden (Bergqist et al. 2021)[22]	NA - focused on app usability
U.S. - TX (ALIVE)[60]	Exercise increased, sedentary time decreased<br>Improvements were greater compared to dietary change intervention group
U.S. - AL, IL (BEAT Cancer)[61,62]	Exercise increased, QOL improved<br>Improvements were greater compared to the control group who received usual care
U.S. - NC, TX (DAMES)[63,64]	Exercise increased, exercise capacity improved<br>Improvements were greater compared to the control group who received weight management information
U.S. - AL, CA, CO, MO (ENERGY)[65-67]	Exercise increased, blood pressure improved, weight decreased<br>Improvements were more favorable in the intervention compared to the control who received a less intensive intervention
U.S. - National (Fit2Thrive)[12-15]	Exercise increased; and, anxiety, depression, fatigue, physical functioning, and sleep-related impairment all improved<br>No control group

© 2025 Wilson OWA et al. *JAMA Network Open*.

Country - Study name or setting	Intervention outcomes and effects
U.S. - CA (I Can!)[68]	NA - Methods only
U.S. - CT (LEAN)[69,70]	Weight decreased, but extra counselling and tracking behavior were no more effective<br>No control group
U.S. - CA (Memory and Motion)[71-73]	Anxiety, depression, fatigue, and physical functioning each improved<br>Improvements in anxiety and physical functioning were greater in the intervention group compared to the waitlist control group
U.S. - NY (Mi Vida Saludable)[18,19]	NA - Methods only
U.S. - TX (MOCHA)[74]	NA - focused on app usability
U.S. - GA, NC (Reach)[30-38]	Physical wellbeing (FACT-B), symptoms, physical functioning (SF-36), fatigue (FACIT-F), and mood (POMS) each improved
U.S. - NJ (Spark People)[75]	Exercise participation, exercise self-efficacy, and social support for exercise did not change over time or differ between groups. Exercise goal setting and planning increased in both groups. Cardiopulmonary fitness and QOL improved. Weight and BMI decreased<br>Only weight and BMI changes differed between the intervention and control group
U.S. - NC (STRENGTH trial)[76]	Exercise, anthropometric characteristics, QOL, and anxiety and depression, and serologic indices did not change
U.S. - DC (The Stepping STONE Study)[77]	Exercise increased, anthropometric variables improved, improved cardiovascular fitness<br>Improvements were greater compared to the control group
U.S. - NC (WELL Body Study)[78]	Energy expenditure, and weight decreased (in group that received information + tracker); the group had less negative perceptions about daily self-weighing
U.S. - NC (WWE)[20,21]	NA - Non-intervention
U.S. - NC, TX (Nyrop et al. 2020)[24]	NA - Non-intervention
U.S. - KS (Fazzino et al. 2017[25]	Exercise was associated with weight change (loss and gain) at different time points<br>There was no control group
U.S. - MA (Ligibel et al. 2010)[79]	Exercise increased, QOL improved, fatigue improved, anthropometric variables did not change<br>There was no control group
U.S. - CT (Park et al. 2016)[80]	Exercise increased<br>Improvements were similar for the standard lifestyle management and targeting the teachable moment groups, which differed from the control group who received standard care
U.S. - MA(Quintiliani et al. 2016)[81]	Exercise and steps increased, weight and fatigue decreased<br>There was no control group

Notes. ALIVE = A Lifestyle Intervention Via Email; DAMES = Daughters and Mothers Against Breast Cancer; ENERGY = Exercise and Nutrition to Enhance Recovery and Good Health for You; I Can! = Improving Cognition After Cancer; MOCHA = Methodist Hospital Cancer Health Application; PHAB = A Home-based Physical Activity Intervention in Breast Cancer Survivors; PROACTIVE = Promoting Physical Activity during Chemotherapy; STRENGTH = Survivor Training for Enhancing Total Health; WSEDI = Web-based self-management exercise and diet intervention; WWE = Walking With Ease; WMFHAB = Weight Management for Health and Beauty; WELL Body = Weighing Every day for Love of Life and Body; MSE = Muscle-strengthening exercise; MPA = Moderate-intensity physical activity; MVPA = Moderate-to-vigorous intensity physical activity; - = Not reported; NA = Not applicable; QOL = Quality of life; BMI = Body mass index

eReferences.

The references below pertain to the content reported within the Supplemental Materials.

1. Page MJ, McKenzie JE, Bossuyt PM, et al. The PRISMA 2020 statement: An updated guideline for reporting systematic reviews. *BMJ*. 2021;372:n71. doi:10.1136/bmj.n71
2. Tricco AC, Lillie E, Zarin W, et al. PRISMA Extension for Scoping Reviews (PRISMA-ScR): Checklist and ExplanationThe PRISMA-ScR Statement. *Ann Intern Med*. 2018;169(7):467-473. doi:10.7326/M18-0850
3. Arksey H, O'Malley L. Scoping studies: Towards a methodological framework. *Int J Soc Res Methodol*. 2005;8(1):19-32. doi:10.1080/1364557032000119616
4. Levac D, Colquhoun H, O'Brien KK. Scoping studies: advancing the methodology. *Implement Sci*. 2010;5(1):69. doi:10.1186/1748-5908-5-69
5. Wilson OWA, Wojcik KM, Rogers LQ, et al. A Scoping Review of Exercise Communication for Breast Cancer Survivors. Published August 28, 2024. osf.io/yxv6w
6. Williamson C, Baker G, Mutrie N, Niven A, Kelly P. Get the message? A scoping review of physical activity messaging. *Int J Behav Nutr Phys Act*. 2020;17(1):51. doi:10.1186/s12966-020-00954-3
7. Covidence systematic review software. Veritas Health Innovation. www.covidence.org
8. Williamson C, Baker G, Tomasone JR, et al. The Physical Activity Messaging Framework (PAMF) and Checklist (PAMC): International consensus statement and user guide. *Int J Behav Nutr Phys Act*. 2021;18(1):164. doi:10.1186/s12966-021-01230-8
9. Lefebvre C, Glanville J, Briscoe S, et al. Searching for and selecting studies. *Cochrane Handbook for Systematic Reviews of Interventions*. 2019:67-107.
10. McKenzie JE, Brennan SE. Chapter 12: Synthesizing and presenting findings using other methods. In: Higgins JPT, Thomas J, Chandler J, Cumpston M, Li T, Page MJ, Welch VA, eds. *Cochrane Handbook for Systematic Reviews of Interventions*. 2024:321-347.
11. Savitz DA, Wise LA, Bond JC, et al. Responding to reviewers and editors about statistical significance testing. *Ann Intern Med*. 2024;177(3):385-386. doi:10.7326/m23-2430 %m 38373303
12. Phillips S, Solk P, Welch W, et al. A technology-based physical activity intervention for patients with metastatic breast cancer (Fit2ThriveMB): Protocol for a randomized controlled trial. *JMIR Res Protoc*. 2021;10(4):e24254. doi:10.2196/24254
13. Phillips SM, Penedo FJ, Collins LM, et al. Optimization of a technology-supported physical activity promotion intervention for breast cancer survivors: Results from Fit2Thrive. *Cancer*. 2022;128(5):1122-1132. doi:10.1002/cncr.34012
14. Welch WA, Solk P, Auster-Gussman L, et al. User-centered development of a smartphone application (Fit2Thrive) to promote physical activity in breast cancer survivors. *Transl Behav Med*. 2022;12(2):203-213. doi:10.1093/tbm/ibab112
15. Phillips SM, McAuley E. Associations between self-reported post-diagnosis physical activity changes, body weight changes, and psychosocial well-being in breast cancer survivors. *Support Care Cancer*. 2015;23(1):159-67. doi:10.1007/s00520-014-2346-5
16. Smith-Turchyn J, McCowan ME, O'Loughlin E, et al. Connecting breast cancer survivors for exercise: protocol for a two-arm randomized controlled trial. *BMC Sports Sci Med Rehabil*. 2021;13:128. doi:10.1186/s13102-021-00341-w
17. Hiensch AE, Monninkhof EM, Schmidt ME, et al. Design of a multinational randomized controlled trial to assess the effects of structured and individualized exercise in patients with metastatic breast cancer on fatigue and quality of life: the EFFECT study. *Trials*. 2022;23(1):610. doi:10.1186/s13063-022-06556-7

© 2025 Wilson OWA et al. *JAMA Network Open*.

18. Contento I, Paul R, Marin-Chollom AM, et al. Developing a diet and physical activity intervention for Hispanic/Latina breast cancer survivors. *Cancer Control*. 2022;29:10732748221133987. doi:10.1177/10732748221133987
19. Santiago-Torres M, Contento I, Koch P, et al. ¡Mi Vida Saludable! A randomized, controlled, 2 × 2 factorial trial of a diet and physical activity intervention among Latina breast cancer survivors: Study design and methods. *Contemp Clin Trials*. 2021;110:106524. doi:10.1016/j.cct.2021.106524
20. Nyrop KA, Callahan LF, Rini C, et al. Adaptation of an evidence-based arthritis program for breast cancer survivors on aromatase inhibitor therapy who experience joint pain. *Prev Chronic Dis*. 2015;12:E91. doi:10.5888/pcd12.140535
21. Nyrop KA, Callahan LF, Rini C, et al. Aromatase inhibitor associated arthralgia: the importance of oncology provider-patient communication about side effects and potential management through physical activity. *Support Care Cancer*. 2016;24(6):2643-50. doi:10.1007/s00520-015-3065-2
22. Bergqvist J, Lundström S, Wengström Y. Patient interactive digital support for women with adjuvant endocrine therapy in order to increase compliance and quality of life. *Support Care Cancer*. 2021;29(1):491-497. doi:10.1007/s00520-020-05476-z
23. Martin E, Di Meglio A, Charles C, et al. Use of mHealth to increase physical activity among breast cancer survivors with fatigue: Qualitative exploration. *JMIR Cancer*. 2021;7(1):e23927. doi:10.2196/23927
24. Nyrop KA, Lee JT, Deal AM, Ki Choi S, Muss HB. Weight-related communications between oncology clinicians and women with obesity at early breast cancer diagnosis: Findings from a review of electronic health records. *Nutr Cancer*. 2020;72(4):576-583. doi:10.1080/01635581.2019.1645863
25. Fazzino TL, Fabian C, Befort CA. Change in physical activity during a weight management intervention for breast cancer survivors: Association with weight outcomes. *Obesity*. 2017;25 Suppl 2(Suppl 2):S109-s115. doi:10.1002/oby.22007
26. Short CE, James EL, Girgis A, D'Souza MI, Plotnikoff RC. Main outcomes of the Move More for Life Trial: A randomised controlled trial examining the effects of tailored-print and targeted-print materials for promoting physical activity among post-treatment breast cancer survivors. *Psychooncology*. 2015;24(7):771-8. doi:10.1002/pon.3639
27. Short CE, James EL, Rebar AL, et al. Designing more engaging computer-tailored physical activity behaviour change interventions for breast cancer survivors: lessons from the iMove More for Life study. *Support Care Cancer*. 2017;25(11):3569-3585. doi:10.1007/s00520-017-3786-5
28. Short CE, Rebar A, James EL, et al. How do different delivery schedules of tailored web-based physical activity advice for breast cancer survivors influence intervention use and efficacy? *J Cancer Surviv*. 2017;11(1):80-91. doi:10.1007/s11764-016-0565-0
29. Vallance JK, Friedenreich CM, Lavallee CM, Culos-Reed N, Mackey JR, Walley B, Courneya KS. Exploring the feasibility of a broad-reach physical activity behavior change intervention for women receiving chemotherapy for breast cancer: A randomized trial. *Cancer Epidemiol Biomarkers Prev*. 2016;25(2):391-8. doi:10.1158/1055-9965.Epi-15-0812
30. Pinto B, Dunsiger S, Stein K. Does a peer-led exercise intervention affect sedentary behavior among breast cancer survivors? *Psychooncology*. 2017;26(11):1907-1913. doi:10.1002/pon.4255
31. Pinto B, Stein K, Dunsiger S. Peer mentorship to promote physical activity among cancer survivors: effects on quality of life. *Psychooncology*. 2015;24(10):1295-1302. doi:10.1002/pon.3884
32. Pinto BM, Dunsiger SI, DeScenza VR, Stein K. Mediators of physical activity outcomes in a peer-led intervention for breast cancer survivors. *Psychooncology*. 2023;32(4):619-627. doi:10.1002/pon.6107

© 2025 Wilson OWA et al. *JAMA Network Open*.

33. Pinto BM, Dunsiger SI, Kindred M, Mitchell S, DeScenza V. Effects of a peer-led physical activity program on psychosocial outcomes among breast cancer survivors. *Psychooncology*. 2024;33(1):e6292. doi:10.1002/pon.6292
34. Pinto BM, Dunsiger SI, Kindred MM, Mitchell S. Physical activity adoption and maintenance among breast cancer survivors: A randomized trial of peer mentoring. *Ann Behav Med*. 2022;56(8):842-855. doi:10.1093/abm/kaab078
35. Pinto BM, Dunsiger SI, Kindred MM, Mitchell S. Peer mentoring for physical activity adoption and maintenance among breast cancer survivors: moderators of physical activity outcomes. *J Cancer Surviv*. 2023;17(4):1211-1220. doi:10.1007/s11764-021-01162-z
36. Pinto BM, Dunsiger SI, Kindred MM, Mitchell S. Mediators of physical activity maintenance during a 12-month randomized controlled trial among breast Cancer survivors. *J Behav Med*. 2023;46(5):745-756. doi:10.1007/s10865-023-00402-0
37. Pinto BM, Rabin C, Abdow S, Papandonatos GD. A pilot study on disseminating physical activity promotion among cancer survivors: a brief report. *Psychooncology*. 2008;17(5):517-21. doi:10.1002/pon.1268
38. Pinto BM, Stein K, Dunsiger S. Peers promoting physical activity among breast cancer survivors: A randomized controlled trial. *Health Psychol*. 2015;34(5):463-72. doi:10.1037/hea0000120
39. Singleton AC, Raeside R, Partridge SR, et al. Supporting women's health outcomes after breast cancer treatment comparing a text message intervention to usual care: the EMPOWER-SMS randomised clinical trial. *J Cancer Surviv*. 2023;17(6):1533-1545. doi:10.1007/s11764-022-01209-9
40. Singleton A, Raeside R, Partridge SR, et al. Co-designing a lifestyle-focused text message intervention for women after breast cancer treatment: Mixed methods study. *J Med Internet Res*. 2021;23(6):e27076. doi:10.2196/27076
41. Reeves MM, Terranova CO, Winkler EAH, et al. Effect of a remotely delivered weight loss intervention in early-stage breast cancer: Randomized controlled trial. *Nutrients*. 2021;13(11)doi:10.3390/nu13114091
42. Spark LC, Fjeldsoe BS, Eakin EG, Reeves MM. Efficacy of a text message-delivered extended contact intervention on maintenance of weight loss, physical activity, and dietary behavior change. *JMIR Mhealth Uhealth*. 2015;3(3):e88. doi:10.2196/mhealth.4114
43. Terranova CO, Winkler EAH, Healy GN, Demark-Wahnefried W, Eakin EG, Reeves MM. Dietary and physical activity changes and adherence to WCRF/AICR cancer prevention recommendations following a remotely delivered weight loss intervention for female breast cancer survivors: The living well after breast cancer randomized controlled trial. *J Acad Nutr Diet*. 2022;122(9):1644-1664.e7. doi:10.1016/j.jand.2022.02.009
44. De Cocker K, Charlier C, Van Hoof E, et al. Development and usability of a computer-tailored pedometer-based physical activity advice for breast cancer survivors. *Eur J Cancer Care*. 2015;24(5):673-82. doi:10.1111/ecc.12225
45. Vallance JK, Courneya KS, Plotnikoff RC, Dinu I, Mackey JR. Maintenance of physical activity in breast cancer survivors after a randomized trial. *Med Sci Sports Exerc*. 2008;40(1):173-80. doi:10.1249/mss.0b013e3181586b41
46. Vallance JK, Courneya KS, Plotnikoff RC, Mackey JR. Analyzing theoretical mechanisms of physical activity behavior change in breast cancer survivors: results from the activity promotion (ACTION) trial. *Ann Behav Med*. 2008;35(2):150-8. doi:10.1007/s12160-008-9019-x
47. Vallance JK, Courneya KS, Plotnikoff RC, Yasui Y, Mackey JR. Randomized controlled trial of the effects of print materials and step pedometers on physical activity and quality of life in breast cancer survivors. *J Clin Oncol*. 2007;25(17):2352-9. doi:10.1200/jco.2006.07.9988

© 2025 Wilson OWA et al. *JAMA Network Open*.

48. Goodwin PJ, Segal RJ, Vallis M, et al. Randomized trial of a telephone-based weight loss intervention in postmenopausal women with breast cancer receiving letrozole: the LISA trial. *J Clin Oncol*. 2014;32(21):2231-9. doi:10.1200/jco.2013.53.1517
49. Goodwin PJ, Segal RJ, Vallis M, et al. The LISA randomized trial of a weight loss intervention in postmenopausal breast cancer. *NPJ Breast Cancer*. 2020;6:6. doi:10.1038/s41523-020-0149-z
50. Zhao FY, Liu JE, Fang XM, et al. Effects of a 12-week exercise-based intervention on weight management in overweight or obese breast cancer survivors: a randomized controlled trial. *Support Care Cancer*. 2024;32(2):98. doi:10.1007/s00520-023-08300-6
51. Lahart IM, Metsios GS, Nevill AM, Kitas GD, Carmichael AR. Randomised controlled trial of a home-based physical activity intervention in breast cancer survivors. *BMC Cancer*. 2016;16:234. doi:10.1186/s12885-016-2258-5
52. Lahart IM, Carmichael AR, Nevill AM, Kitas GD, Metsios GS. The effects of a home-based physical activity intervention on cardiorespiratory fitness in breast cancer survivors; a randomised controlled trial. *J Sports Sci*. 2018;36(10):1077-1086. doi:10.1080/02640414.2017.1356025
53. Falzon C, Radel R, Cantor A, d'Arripe-Longueville F. Understanding narrative effects in physical activity promotion: The influence of breast cancer survivor testimony on exercise beliefs, self-efficacy, and intention in breast cancer patients. *Support Care Cancer*. 2015;23(3):761-8. doi:10.1007/s00520-014-2422-x
54. Kong S, Lee JK, Kang D, et al. Comparing the effectiveness of a wearable activity tracker in addition to counseling and counseling only to reinforce leisure-time physical activity among breast cancer patients: A randomized controlled trial. *Cancers*. 2021;13(11)doi:10.3390/cancers13112692
55. Lee H, Uhm KE, Cheong IY, et al. Patient satisfaction with mobile health (mHealth) application for exercise intervention in breast cancer survivors. *J Med Syst*. 2018;42(12):254. doi:10.1007/s10916-018-1096-1
56. Park SW, Lee I, Kim JI, et al. Factors associated with physical activity of breast cancer patients participating in exercise intervention. *Support Care Cancer*. 2019;27(5):1747-1754. doi:10.1007/s00520-018-4427-3
57. Lee MK, Yun YH, Park HA, Lee ES, Jung KH, Noh DY. A Web-based self-management exercise and diet intervention for breast cancer survivors: pilot randomized controlled trial. *Int J Nurs Stud*. 2014;51(12):1557-67. doi:10.1016/j.ijnurstu.2014.04.012
58. Travier N, Buckland G, Vendrell JJ, et al. Changes in metabolic risk, insulin resistance, leptin and adiponectin following a lifestyle intervention in overweight and obese breast cancer survivors. *Eur J Cancer Care*. 2018;27(4):e12861. doi:10.1111/ecc.12861
59. Travier N, Fonseca-Nunes A, Javierre C, et al. Effect of a diet and physical activity intervention on body weight and nutritional patterns in overweight and obese breast cancer survivors. *Med Oncol*. 2014;31(1):783. doi:10.1007/s12032-013-0783-5
60. Paxton RJ, Hajek R, Newcomb P, et al. A lifestyle intervention via email in minority breast cancer survivors: Randomized parallel-group feasibility study. *JMIR Cancer*. 2017;3(2):e13. doi:10.2196/cancer.7495
61. Rogers LQ, McAuley E, Anton PM, et al. Better exercise adherence after treatment for cancer (BEAT Cancer) study: Rationale, design, and methods. *Contemp Clin Trials*. 2012;33(1):124-37. doi:10.1016/j.cct.2011.09.004
62. Rogers LQ, Courneya KS, Anton PM, et al. Effects of the BEAT Cancer physical activity behavior change intervention on physical activity, aerobic fitness, and quality of life in breast cancer survivors: a multicenter randomized controlled trial. *Breast Cancer Res Treat*. 2015;149(1):109-19. doi:10.1007/s10549-014-3216-z
63. Demark-Wahnefried W, Jones LW, Snyder DC, et al. Daughters and Mothers Against Breast Cancer (DAMES): Main outcomes of a randomized controlled trial of weight loss in

overweight mothers with breast cancer and their overweight daughters. *Cancer*. 2014;120(16):2522-34. doi:10.1002/cncr.28761
64. Tometich DB, Mosher CE, Winger JG, Badr HJ, Snyder DC, Sloane RJ, Demark-Wahnefried W. Effects of diet and exercise on weight-related outcomes for breast cancer survivors and their adult daughters: an analysis of the DAMES trial. *Support Care Cancer*. 2017;25(8):2559-2568. doi:10.1007/s00520-017-3665-0
65. Rock CL, Byers TE, Colditz GA, et al. Reducing breast cancer recurrence with weight loss, a vanguard trial: The Exercise and Nutrition to Enhance Recovery and Good Health for You (ENERGY) Trial. *Contemp Clin Trials*. 2013;34(2):282-95. doi:10.1016/j.cct.2012.12.003
66. Kenzik KM, Demark-Wahnefried W, Ganz PA, Colditz G, Rock CL, Rogers LQ. Changes in body mass index and physical activity predict changes in vitality during a weight loss trial in breast cancer survivors. *Ann Behav Med*. 2018;52(12):999-1009. doi:10.1093/abm/kay004
67. Rock CL, Flatt SW, Byers TE, et al. Results of the Exercise and Nutrition to Enhance Recovery and Good Health for You (ENERGY) trial: A behavioral weight loss intervention in overweight or obese breast cancer survivors. *J Clin Oncol*. 2015;33(28):3169-76. doi:10.1200/jco.2015.61.1095
68. Hartman SJ, Weiner LS, Natarajan L, et al. A randomized trial of physical activity for cognitive functioning in breast cancer survivors: Rationale and study design of I Can! Improving Cognition After Cancer. *Contemp Clin Trials*. 2021;102:106289. doi:10.1016/j.cct.2021.106289
69. Lisevick A, Cartmel B, Harrigan M, et al. Effect of the Lifestyle, Exercise, and Nutrition (LEAN) study on long-term weight loss maintenance in women with breast cancer. *Nutrients*. 2021;13(9)doi:10.3390/nu13093265
70. Harrigan M, Cartmel B, Loftfield E, et al. Randomized trial comparing telephone versus in-person weight loss counseling on body composition and circulating biomarkers in women treated for breast cancer: The Lifestyle, Exercise, and Nutrition (LEAN) study. *J Clin Oncol*. 2016;34(7):669-76. doi:10.1200/jco.2015.61.6375
71. Hartman SJ, Weiner LS, Nelson SH, et al. Mediators of a physical activity intervention on cognition in breast cancer survivors: Evidence from a randomized controlled trial. *JMIR Cancer*. 2019;5(2):e13150. doi:10.2196/13150
72. Hartman SJ, Natarajan L, Palmer BW, Parker B, Patterson RE, Sears DD. Impact of increasing physical activity on cognitive functioning in breast cancer survivors: Rationale and study design of Memory & Motion. *Contemp Clin Trials*. 2015;45(Pt B):371-376. doi:10.1016/j.cct.2015.09.021
73. Hartman SJ, Nelson SH, Myers E, et al. Randomized controlled trial of increasing physical activity on objectively measured and self-reported cognitive functioning among breast cancer survivors: The memory & motion study. *Cancer*. 2018;124(1):192-202. doi:10.1002/cncr.30987
74. Stubbins R, He T, Yu X, et al. A behavior-modification, clinical-grade mobile application to improve breast cancer survivors' accountability and health outcomes. *JCO Clin Cancer Inform*. 2018;2:1-11. doi:10.1200/cci.18.00054
75. Ferrante JM, Devine KA, Bator A, Rodgers A, Ohman-Strickland PA, Bandera EV, Hwang KO. Feasibility and potential efficacy of commercial mHealth/eHealth tools for weight loss in African American breast cancer survivors: pilot randomized controlled trial. *Transl Behav Med*. 2020;10(4):938-948. doi:10.1093/tbm/iby124
76. Demark-Wahnefried W, Case LD, Blackwell K, et al. Results of a diet/exercise feasibility trial to prevent adverse body composition change in breast cancer patients on adjuvant chemotherapy. *Clin Breast Cancer*. 2008;8(1):70-9. doi:10.3816/CBC.2008.n.005
77. Sheppard VB, Hicks J, Makambi K, Hurtado-de-Mendoza A, Demark-Wahnefried W, Adams-Campbell L. The feasibility and acceptability of a diet and exercise trial in overweight and obese black breast cancer survivors: The Stepping STONE study. *Contemp Clin Trials*. 2016;46:106-113. doi:10.1016/j.cct.2015.12.005

78. Valle CG, Deal AM, Tate DF. Preventing weight gain in African American breast cancer survivors using smart scales and activity trackers: a randomized controlled pilot study. *J Cancer Surviv*. 2017;11(1):133-148. doi:10.1007/s11764-016-0571-2
79. Ligibel JA, Partridge A, Giobbie-Hurder A, Campbell N, Shockro L, Salinardi T, Winer EP. Physical and psychological outcomes among women in a telephone-based exercise intervention during adjuvant therapy for early stage breast cancer. *J Womens Health*. 2010;19(8):1553-9. doi:10.1089/jwh.2009.1760
80. Park CL, Cho D, Salner AL, Dornelas E. A randomized controlled trial of two mail-based lifestyle interventions for breast cancer survivors. *Support Care Cancer*. 2016;24(7):3037-46. doi:10.1007/s00520-016-3129-y
81. Quintiliani LM, Mann DM, Puputti M, Quinn E, Bowen DJ. Pilot and feasibility test of a mobile health-supported behavioral counseling intervention for weight management among breast cancer survivors. *JMIR Cancer*. 2016;2(1)doi:10.2196/cancer.5305
82. Donnelly JE, Blair SN, Jakicic JM, Manore MM, Rankin JW, Smith BK. American College of Sports Medicine position stand. Appropriate physical activity intervention strategies for weight loss and prevention of weight regain for adults. *Med Sci Sports Exerc*. 2009;41(2):459-71. doi:10.1249/MSS.0b013e3181949333
83. World Cancer Research Fund/American Institute for Cancer Research. Diet, nutrition, physical activity and cancer: A Global Perspective. 2018;
84. Australian Government Department of Health. *Australia's Physical Activity and Sedentary Behaviour Guidelines 2014*. 2014. https://www.health.gov.au/topics/physical-activity-and-exercise/physical-activity-and-exercise-guidelines-for-all-australians
85. Vallance JK, Courneya KS, Taylor LM, Plotnikoff RC, Mackey JR. Development and evaluation of a theory-based physical activity guidebook for breast cancer survivors. *Health Educ Behav*. 2008;35(2):174-89. doi:10.1177/1090198106287693
86. Campbell KL, Winters-Stone KM, Wiskemann J, et al. Exercise guidelines for cancer survivors: Consensus statement from international multidisciplinary roundtable. *Med Sci Sports Exerc*. 2019;51(11):2375-2390. doi:10.1249/mss.0000000000002116
87. Schmitz KH, Courneya KS, Matthews C, et al. American College of Sports Medicine roundtable on exercise guidelines for cancer survivors. *Med Sci Sports Exerc*. 2010;42(7):1409-26. doi:10.1249/MSS.0b013e3181e0c112
88. Segal R, Zwaal C, Green E, Tomasone JR, Loblaw A, Petrella T. Exercise for people with cancer: A clinical practice guideline. *Curr Oncol*. 2017;24(1):40-46. doi:10.3747/co.24.3376
89. Bull FC, Experts Working Groups. *Physical Activity Guidelines in the UK: Review and recommendations*. 2010. https://www.gov.uk/government/uploads/system/uploads/attachment_data/file/213743/dh_128255.pdf.
90. Garber CE, Blissmer B, Deschenes MR, et al. American College of Sports Medicine position stand. Quantity and quality of exercise for developing and maintaining cardiorespiratory, musculoskeletal, and neuromotor fitness in apparently healthy adults: guidance for prescribing exercise. *Med Sci Sports Exerc*. 2011;43(7):1334-59. doi:10.1249/MSS.0b013e318213fefb
91. Doyle C, Kushi LH, Byers T, et al. Nutrition and physical activity during and after cancer treatment: An American Cancer Society guide for Informed choices. *CA Cancer J Clin*. 2006;56(6):323-353. doi:10.3322/canjclin.56.6.323
92. Rock CL, Doyle C, Demark-Wahnefried W, et al. Nutrition and physical activity guidelines for cancer survivors. *CA Cancer J Clin*. 2012;62(4):242-274. doi:10.3322/caac.21142
93. Clinton SK, Giovannucci EL, Hursting SD. The World Cancer Research Fund/American Institute for Cancer Research Third Expert Report on Diet, Nutrition, Physical Activity, and Cancer: Impact and Future Directions. *J Nutr*. 2020;150(4):663-671. doi:10.1093/jn/nxz268

© 2025 Wilson OWA et al. *JAMA Network Open*.

94. Jensen MD, Ryan DH, Apovian CM, et al. 2013 AHA/ACC/TOS guideline for the management of overweight and obesity in adults: a report of the American College of Cardiology/American Heart Association Task Force on Practice Guidelines and The Obesity Society. *Circulation*. 2014;129(25 Suppl 2):S102-38. doi:10.1161/01.cir.0000437739.71477.ee
95. Abernethy PJ, Jürimäe J, Logan PA, Taylor AW, Thayer RE. Acute and chronic response of skeletal muscle to resistance exercise. *Sports Med*. 1994;17(1):22-38. doi:10.2165/00007256-199417010-00003
96. Ballard-Barbash R, Hunsberger S, Alciati MH, et al. Physical activity, weight control, and breast cancer risk and survival: clinical trial rationale and design considerations. *J Natl Cancer Inst*. 2009;101(9):630-43. doi:10.1093/jnci/djp068

© 2025 Wilson OWA et al. *JAMA Network Open*.
