# Supplementary material for: Exercise Communication for Breast Cancer Survivors: A Systematic Scoping Review
Source: JAMA Netw Open. 2025 May 16;8(5):e258862. doi: 10.1001/jamanetworkopen.2025.8862 (PMC12084847; doi:10.1001/jamanetworkopen.2025.8862)
Supplement: Supplement 2. — Data Sharing Statement [file jamanetwopen-e258862-s002.pdf]

Data Sharing Statement

Data
Data available: No

Additional Information
Explanation for why data not available: Data sharing is not applicable to this article as no datasets were analyzed or generated during the current study. All studies summarized in this scoping review are listed in the data supplement.
